# Multifeature quantitative motor assessment of upper limb ataxia including drawing and reaching

**DOI:** 10.1002/acn3.52024

**Published:** 2024-04-08

**Authors:** Dominik Hermle, Robin Schubert, Pascal Barallon, Winfried Ilg, Rebecca Schüle, Ralf Reilmann, Matthis Synofzik, Andreas Traschütz

**Affiliations:** ^1^ Division Translational Genomics of Neurodegenerative Diseases, Hertie Institute for Clinical Brain Research and Center of Neurology University of Tübingen Tübingen Germany; ^2^ George‐Huntington‐Institute Münster Germany; ^3^ Section Computational Sensomotorics, Hertie Institute for Clinical Brain Research Tübingen Germany; ^4^ Centre for Integrative Neuroscience (CIN) Tübingen Germany; ^5^ German Center for Neurodegenerative Diseases (DZNE) Tübingen Germany; ^6^ Division of Neurodegenerative Disease, Department of Neurology Heidelberg University Hospital Heidelberg Germany; ^7^ Department of Neurodegenerative Diseases Center of Neurology and Hertie Institute for Clinical Brain Research, University of Tübingen Tübingen Germany; ^8^ Department of Clinical Radiology University of Münster Münster Germany

## Abstract

**Objective:**

Voluntary upper limb movements are an ecologically important yet insufficiently explored digital‐motor outcome domain for trials in degenerative ataxia. We extended and validated the trial‐ready quantitative motor assessment battery “Q‐Motor” for upper limb movements with clinician‐reported, patient‐focused, and performance outcomes of ataxia.

**Methods:**

Exploratory single‐center cross‐sectional assessment in 94 subjects (46 cross‐genotype ataxia patients; 48 matched controls), comprising five tasks measured by force transducer and/or position field: Finger Tapping, diadochokinesia, grip‐lift, and—as novel implementations—Spiral Drawing, and Target Reaching. Digital‐motor measures were selected if they discriminated from controls (AUC >0.7) and correlated—with at least one strong correlation (rho ≥0.6)—to the Scale for the Assessment and Rating of Ataxia (SARA), activities of daily living (FARS‐ADL), and the Nine‐Hole Peg Test (9HPT).

**Results:**

Six movement features with 69 measures met selection criteria, including *speed* and *variability* in all tasks, *stability* in grip‐lift, and *efficiency* in Target Reaching. The novel drawing/reaching tasks best captured impairment in dexterity (|rho_9HPT_| ≤0.81) and FARS‐ADL upper limb items (|rho_ADLul_| ≤0.64), particularly by kinematic analysis of *smoothness (SPARC).* Target *hit rate*, a composite of *speed* and *endpoint precision*, almost perfectly discriminated ataxia and controls (AUC: 0.97). Selected measures in all tasks discriminated between mild, moderate, and severe impairment (SARA upper limb composite: 0–2/>2–4/>4–6) and correlated with severity in the trial‐relevant mild ataxia stage (SARA ≤10, *n* = 20).

**Interpretation:**

Q‐Motor assessment captures multiple features of impaired upper limb movements in degenerative ataxia. Validation with key clinical outcome domains provides the basis for evaluation in longitudinal studies and clinical trial settings.

## Introduction

Cerebellar ataxias are neurodegenerative diseases of the cerebellum and its projections, causing progressive impairment of balance, voluntary movements, and speech. With molecular treatments on the horizon for the first genetic ataxias, sensitive outcome measures are now urgently needed to conduct sufficiently powered interventional trials.^1^ While the sensitivity of clinical outcome assessments for the severity and progression of ataxia is limited,^2^, ^3^, ^4^ digital‐motor assessments have been shown to provide more objective, reliable, and longitudinally responsive outcome measures, particularly for the impairment of gait and stance.^5^, ^6^, ^7^, ^8^

Compared to the gait and stance domain, digital‐motor assessment of upper limb movements has been much less studied in ataxias. Moreover, without a consensus on how to best measure (the construct of) “appendicular ataxia,” very different technologies, motor tasks, and conceptual frameworks have been used.^9^, ^10^, ^11^, ^12^, ^13^, ^14^ Within the *International Classification of Functioning, Disability and Health (ICF)*, appendicular ataxia is related to the “control of voluntary movement functions,” including coordination of “simple, complex, and visually directed movements,” which enable activities of domestic life and self‐care such as washing oneself, caring for body parts, toileting, dressing, eating, and drinking.^15^ Impairment in these activities is core element of patient‐focused functional ataxia scales such as the Activities of Daily Living part of the Friedreich Ataxia Rating Scale (FARS‐ADL).^16^ In a recently developed patient‐reported outcome measure of ataxia (PROM‐ataxia), even one‐third of physical and ADL items address impairment of upper limb movements.^17^ In addition to functional relevance, digital‐motor assessment in the upper limb domain might also meet two pragmatic needs for clinical ataxia trials: First, it may outperform the relatively poor sensitivity of current clinician‐reported outcomes (ClinROs)—such as the Scale for the Assessment and Rating of Ataxia (SARA)—and even clinical performance outcomes (PerfOs) such as the Nine‐Hole Peg Test (9HPT) in this domain.^2^, ^3^, ^5^, ^18^, ^19^ Second, specifically upper limb digital‐motor assessments may be applicable across all disease stages, including both the advanced stage—when loss of free ambulation hampers clinical or digital motor assessment of gait and stance—and particularly also the most trial‐relevant mild stage before clinician‐reported assessments become responsive to appendicular ataxia.^2^, ^20^

Digital‐motor assessment of voluntary movements has been particularly successful in Huntington's disease, where the integrated trial‐ready quantitative motor system “Q‐Motor” has not only been applied in large international natural history studies,^21^ but even used as standardized endpoint in multicenter clinical trials, recognized by regulatory agencies as a clinical endpoint.^22^, ^23^ Q‐Motor is a laboratory‐/clinic‐based assessment including standardized upper limb motor tasks also suitable to capture cerebellar pathophysiology.^24^ Specifically, we here hypothesized that its Finger Tapping (digitomotography) and diadochokinesia (dysdiadochomotography) tasks would be able to capture cerebellar deficits in speed and rhythmicity, and that its grip‐lift task (manumotography and hyperkinesiomotography) would capture cerebellar deficits in limb stability and force control.^25^, ^26^ For a comprehensive assessment of appendicular ataxia, however, the so far existing Q‐Motor battery^21^ lacked a motor task that probes kinematic deficits such as smoothness or efficiency of upper limb movements.^27^ Thus, we here also extended the battery by two novel ataxia tasks: (i) Spiral Drawing as a complex fine motor task that has long been used as part of the International Cooperative Ataxia Rating Scale (ICARS),^28^ but also regained attention in current interventional trials^29^ and digital assessments protocols^30^; and (ii) sequential Target Reaching, which requires a visually directed multi‐joint reaching and pointing movement susceptible to irregularity, decomposition, dysmetria, and kinetic tremor in ataxia.^31^, ^32^

This exploratory study provides a cross‐sectional validation of this extended quantitative motor assessment of upper limb movements in a large single‐center cross‐genotype cohort of degenerative ataxias, correlating its digital motor outcome measures against all key clinical outcome assessments (COAs) of appendicular ataxia, including clinician‐reported, patient‐focused, and performance outcomes of ataxia severity. Overall, we show that quantitative motor assessment (i) captures multiple features of voluntary upper limb movements with valid outcome measures; that it can (ii) discriminate between different severity levels of upper limb ataxia; and that (iii) its tasks and measures are sensitive to impairment even in the trial‐relevant mild ataxia stage.

## Methods

### Cohort and clinical outcome assessment

The study cohort was recruited from consecutive patients presenting to the ataxia clinic of the University Hospital Tübingen between February 2020 and October 2021. Patients had been eligible if they had degenerative cerebellar ataxia with or without sensory ataxia (see Supplement 1 for list of diagnoses). Patients with additional non‐cerebellar motor or cognitive involvement common to genetic ataxias (e.g., Parkinsonism, hyperkinetic movement disorders, pyramidal tract involvement, and dementia) were excluded if this involvement was severe enough to cause relevant additional impairment of upper limb movement, as rated by the ataxia expert (M.S. and A.T.). Clinical assessments comprised (i) demographics (age, sex, height, and weight), (ii) handedness as determined by the short form of the Edinburgh Handedness Inventory,^33^ (iii) the SARA^34^ as clinician‐reported outcome, (iv) the FARS‐ADL^16^ by interview, as patient‐focused outcome, and (v) the 9HPT (version Rolyan®) as performance outcome of ataxia. Except for eleven controls with dominant hand testing only, the 9HPT was applied to the dominant and nondominant hand. Performance in the 9HPT was calculated as the mean duration of two trials per hand, and then averaged between dominant and nondominant hand if applicable. Age‐ and sex‐matched healthy controls were recruited among patients' company, staff members, and medical students. This study was approved by the Institutional Review Board of the Medical Faculty of the University of Tübingen (824/2019BO2), and all subjects provided written informed consent.

### Quantitative motor assessment

#### Adaptation of setup and established tasks

**Figure 1:**
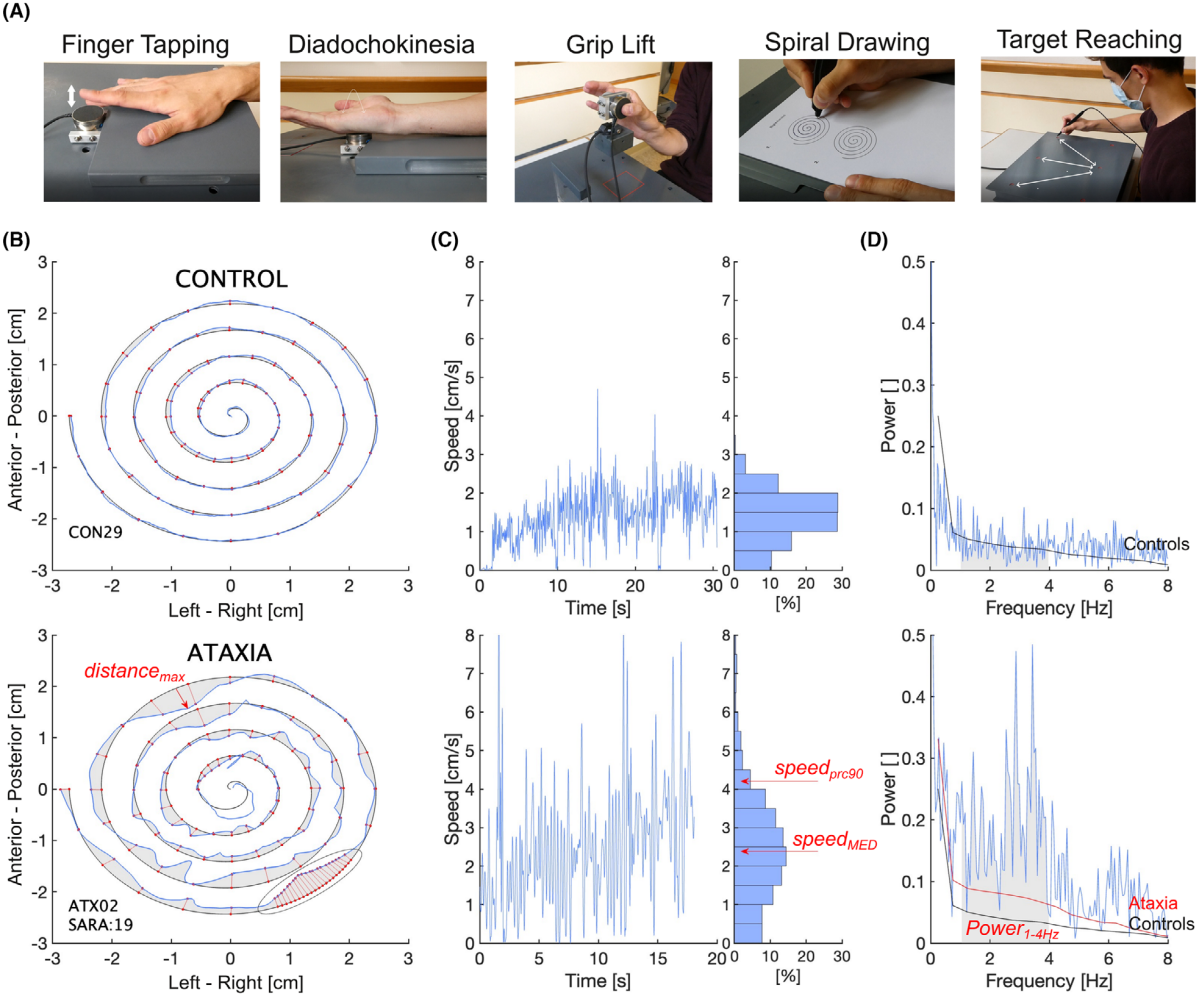
Illustration of tasks (A) and quantitative analysis of the novel Spiral Drawing task in a representative healthy control and ataxia patient (B–D). (B) Spatial measures such as the maximum error (*distance*_*max*_) were calculated based on the distance between points of the same angle on template and spiral trace (red lines), after transformation of data into polar coordinates and sampling of distances at 1000 angles (see highlighted sector for actual resolution). (C) Spatiotemporal measures were calculated based on the distribution of instantaneous values across the trial duration, for example as median, median absolute deviation (MAD), or highest decile of drawing speeds. Note the unstable and fast speeds in ataxia, while trial duration and absolute speed were effectively arbitrary due to the self‐paced task execution. (D) Frequency spectrum of time‐speed series, demonstrating increased low‐frequency power in ataxia patients as compared to controls, both in representative examples and population averages (red and black curves). Power between 1 and 4 Hz (shaded areas) showed the largest difference between ataxia and controls, and was extracted as distinct smoothness measure.

Quantitative motor assessment was performed with a revised Q‐Motor setup and built‐in QmedX software (Fig. 1A; Q‐Motor 2.0, George‐Huntington‐Institute and QuantiMedis, Münster, Germany). Supplement 2 provides details of the setup, tasks, and measures. In short, subjects were first assessed using three established upper limb motor tasks previously described in detail, specifically speeded Finger Tapping (digitomotography) and diadochokinesia (dysdiadochomotography), and a grip‐lift task (manumotography and hyperkinesiomotography).^21^ All measures were averaged over three successive trials for each the dominant and nondominant hand.

#### Spiral Drawing

In this novel fine motor task, subjects were required to trace a 5‐cm diameter Archimedes spiral^35^ on a paper template from the inside out using a digitizer pen (Polhemus FASTRAK, Polhemus Inc., Colchester, VT) with an attached pencil lead (Fig. 1A,B). With their forearms resting on the table, thus aiming for ecological task conditions, subjects were instructed to trace the template “as accurately as possible” (i.e., irrespective of duration) and “as smoothly as possible” (i.e., without intermittent lift‐offs and pauses). Each subject performed two trials (practice trial, and test trial used for analysis) with their dominant hand, after familiarization with the digitizer pen by writing their name.

Spatial measures of Spiral Drawing were extracted based on a transformation of positional data into polar coordinates and a fit to the digital copy of the paper template (see Fig. 1B for illustrative example of control and ataxia patient; and Supplement 2 for detailed methods). The distances between the subject's trace and the template at matched angles were sampled at 1000 angular phases (corresponding to an angular resolution of 1.8° for five windings of 360°), and then used to calculate their cumulative sum (*distance*_*total*_), median (*distance*_*MED*_) and median absolute deviation (*distance*_*MAD*_), maximum (*distance*_*max*_), 90th percentile (*distance*_*prc90*_), and cumulative sum across the highest decile (*distance*_*prc90‐100*_).

Measures of Spiral Drawing in the spatiotemporal and frequency domain were extracted based on the first (i.e., *speed*) and second (i.e., acceleration, *acc*) temporal derivative of the positional data, each followed by digital filtering with a fourth‐order 8 Hz Butterworth filter. The distribution of instantaneous speeds (or absolute values of acceleration) across the trial duration was used to calculate the respective median, median absolute deviation, 90th percentile, and cumulative sum across the highest decile (e.g., *speed*_*MED*_, *speed*_*MAD*_, *speed*_*prc90*_, and *speed*_*prc90‐100*_), given that speed and acceleration appeared to be skewed toward higher values in ataxia patients (Fig. 1C). Fast Fourier transform analysis (FFT) was applied to calculate smoothness of movement by means of the Spectral Arc Length (*SPARC*) of the power spectrum of the time‐speed series, based on open‐source algorithms.^36^ In addition, manual comparison of the power spectrum between ataxia patients and healthy controls revealed a consistent peak of power in ataxia patients between 1 and 4 Hz (Fig. 1D). Thus, the cumulative power in this narrow frequency band (*Power*_*1‐4Hz*_) was calculated as an additional smoothness measure in the frequency domain.

#### Target Reaching

**Figure 2:**
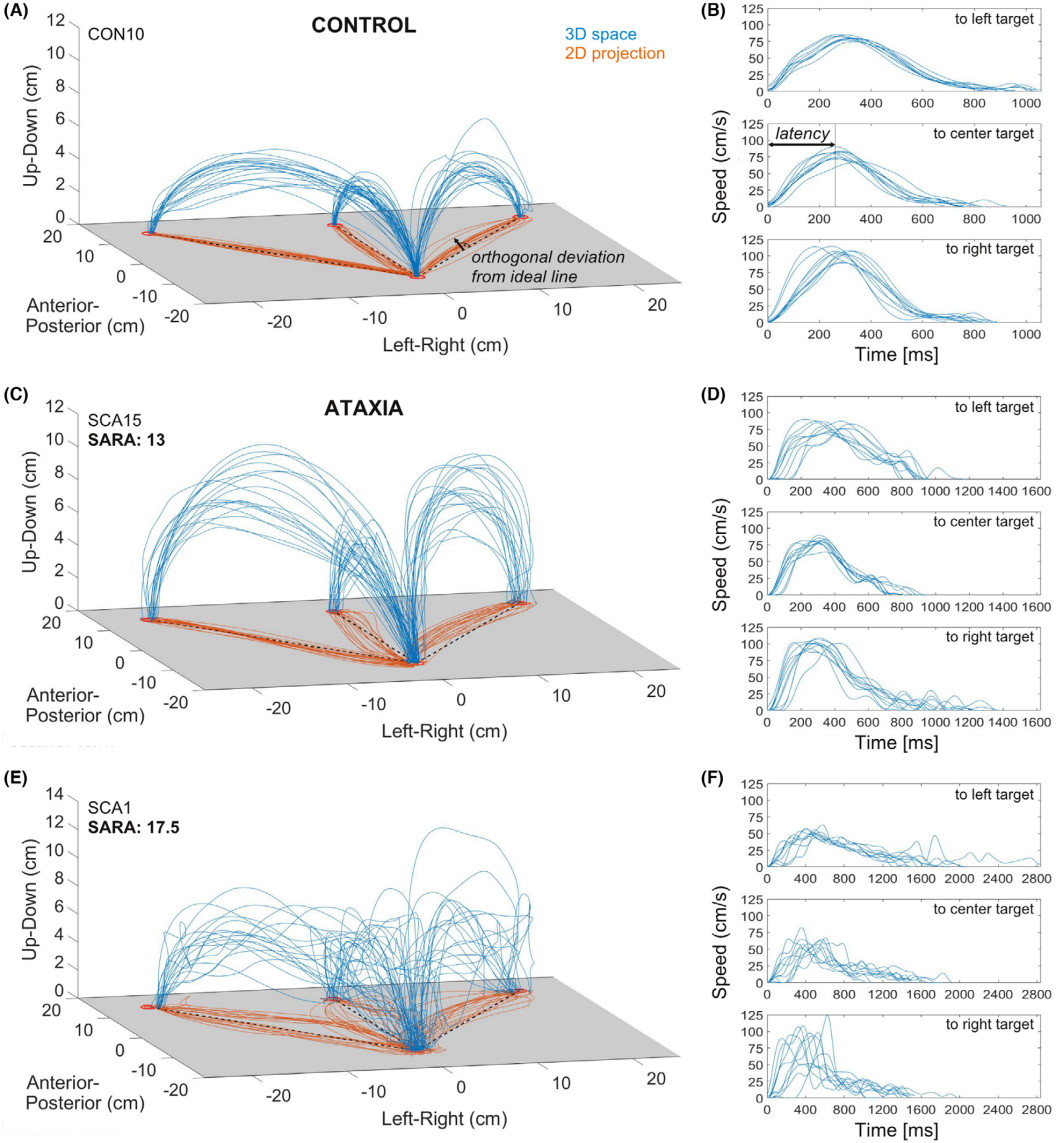
Illustration and analysis of the novel Target Reaching task, demonstrating the increased between‐movement variability and within‐movement irregularity in ataxia. (A, C, and E) Spatial trajectory of repeated movements within one trial in a healthy control and two representative ataxia patients. All trajectories were analyzed in the actual 3D space (blue curves) as well as virtual projections onto the 2D plane of the board (orange). Measures for the amplitude of kinetic tremor were calculated based on the orthogonal deviation from the ideal line between two targets in the 2D plane (dashed lines). (B, D, and F) Time‐speed series of repeated centrifugal movements to one of the three targets. The *latency* between onset and maximum speed was calculated to measure variability in timing for one specific point on the curve. Heterogeneity between the full time‐speed series was also estimated using dynamic time warp (see main text).

In this novel visually directed movement task, subjects were required to perform an ordered sequence of multi‐joint reaching and pointing movements.^37^ With a handheld digitizer stylus (FASTRAK Digitizer, Polhemus Inc., Colchester, VT), subjects were instructed to point—“as fast and accurately as possible”—alternately between four red circular targets of 1.5 cm diameter (Figs. 1A and 2A,C,E; see Supplement 2 for detailed methods). Each subject performed two trials (practice trial, and test trial used for analysis) with 10 reaching movements per target and direction. After segmentation of each trial into the six different reaching directions, movements were separately analyzed in four complementary domains:

- Temporal: *Frequency* was calculated as the number of taps per second, regardless of whether the target was hit. The *inter‐tap interval (ITI)* was calculated as the time between the onset of two consecutive taps, defined by reaching the minimum vertical position. *Tap duration (TD)* was determined as the interval the stylus remained at the minimum vertical position.
- Spatial: Based on the *distance* of the tap position from the target center in the 2D plane of the board, individual pointing movements were classified as ‘hits’ when the distance was smaller than the target radius, and a corresponding *hit rate* was defined by the number of hits per second of trial. A measure *dysmetria* was calculated as the largest deflection from the target center in the anterior–posterior dimension prior to the tap. The *path* length of the movement trajectory from one target to the next was calculated for the actual trajectory in 3D space (*path*_*3D*_), and separately for each individual Cartesian dimension (*path*_*AP*_ = anterior–posterior; *path*_*LR*_ = left–right; *path*_*V*_ = vertical). To exclude the gross movement component with and against gravity in the vertical dimension, path length was also calculated for the 2D projection of the trajectory on the board (*path*_*2D*_), as if it was a planar reaching movement (see Fig. 2A,C,E for illustrative examples). To capture kinetic tremor, this 2D projection was also used to calculate a virtual movement vector orthogonal to the ideal line between two targets (see Fig. 2A for illustration). The maximum of this orthogonal vector (*deviation*_*max*_) was defined as measure estimating of the amplitude of kinetic tremor. The cumulative sum (*deviation*_*total*_) of this orthogonal vector was defined as a composite measure combining kinetic tremor and speed of movement.
- Spatiotemporal: For both the actual 3D trajectory and its 2D projection, we calculated the first (i.e., speed) and second (i.e., acceleration) temporal derivative, each followed by digital filtering with a fourth order, 8 Hz Butterworth filter. For each direction and repetition of movement, the respective time segments for *speed*, *acceleration* (*acc*, i.e., positive acceleration), and *deceleration* (*dec*, i.e., negative acceleration) were used to calculate their respective mean and maximum, as well as the corresponding *latency* until their maximum (e.g., *speed*_*mean*_, *speed*_*max*_, and *latency*_*speed*_; see Fig. 2B,D,F for illustrative examples). The similarity (or heterogeneity) between time‐speed series of the same movement was quantified using *dynamic time warp (dtw)*, which calculates the sum of Euclidean distances between two curves after optimal alignment in time. Specifically, *dtw* was calculated as the median of all possible pairwise comparisons between repetitions of each movement direction.
- Frequency: FFT was applied to calculate smoothness of 3D and 2D movement trajectories by means of the Spectral Arc Length (*SPARC*) of the power spectrum of the time‐speed series, based on open‐source algorithms.^36^

All Target Reaching measures were calculated as *median (MED)* across repetitions of the same movement, which is more robust than the mean against outliers due to non‐systematic aberrant behavior. Accordingly, the *median absolute deviation (MAD)* was calculated as robust measure of variability across repetitions where applicable, for example, as *path*_*3D,MAD*_ or *latency*_*speed,MAD*_. For the purpose of this validation study, all measures were ultimately averaged across the six movement directions.

### Selection of measures

To handle redundancy of overall 188 measures across all tasks, and as a conceptual framework for their interpretability, measures were grouped by seven higher order movement features comprising (i) *speed*, (ii) *variability*, that is, fluctuation between repetitions of the same movement, (iii) *efficiency*, that is, deviation from the ideal movement trajectory, (iv) kinematic *smoothness* of the movement, (v) *endpoint precision*, and (vi) positional *stability*, that is, unvoluntary movement during stationary lifting, as well as (vii) *force control* (see Supplement 3 for matrix of measures and rationale for features).^26^, ^27^ Within these movement features, measures were selected based on a systematic filter of validation criteria considering key clinical outcome domains for ataxia severity. Specifically, this filter combined (i) discrimination of ataxia patients from healthy controls with an AUC >0.7 in a receiver operating characteristic (ROC) analysis^38^ (known‐groups validity), and (ii) significant Spearman correlations to the SARA as clinician‐reported outcome,^34^ the FARS‐ADL as patient‐focused outcome,^16^ and the 9HPT as performance outcome^19^ (convergent validity), and (iii) at least one strong correlation to these three outcome measures (rho ≥0.6). Correlations to the specific subscale of upper limb items of the SARA (rho_SARAul_, i.e., items nose‐finger, finger‐chase, and alternating hand movements) and FARS‐ADL (rho_ADLul_, i.e., items cutting/handling, dressing, and hygiene) were considered equivalent to correlations with the full scale.

### Statistical analysis

All descriptive and statistical tests were performed using MatLab R2022b (The MathWorks, Natick, MA). Descriptive statistics are reported as mean ± standard deviation, or median and interquartile range (IQR). Demographic data were compared between ataxia patients and controls using t‐test (for age as continuous data) and Fisher's exact test (for sex as categorical variable). To examine discrimination between different severity levels of upper limb ataxia, the SARA upper limb composite (range: 0–12 points) was used to split the full cohort into three groups of mild (SARA_ul_: 0–2 points), moderate (>2–4 points), and severe (>4–6 points) upper limb ataxia. Sensitivity in the mild ataxia stage was analyzed in a subcohort of patients defined by a total SARA≤10 points^2^. Spearman correlation (to COAs or demographic data), ROC analysis (between ataxia patients and controls), as was well as the Wilcoxon rank‐sum test and Kruskal–Wallis test (for comparisons between two and three severity levels, respectively) were applied as non‐parametric methods to account for non‐normal distributions of measures. Spearman correlation also accounted for variable non‐linear associations to COAs. Because of the redundancy of measures within feature domains (e.g., six measures for tapping speed) and their complementary testing in both hands, statistical results were not fully independent between measures. In addition, selection of measures required significant correlations to all COAs at once, unlikely to co‐occur by chance. Thus, the significance level for correlations was not adjusted for multiple comparisons in this exploratory study. Across all tests, significance levels are reported at levels of *p* < 0.05 (*) and *p* < 0.01 (**), and *p* < 0.001 (***). Correlations are referred to as “very strong” (rho: 0.80–1.0), “strong” (0.60–0.79), “moderate” (0.40–0.59), and “weak” (0.20–0.39). Unless reported in the main text, results for all selected measures including their correlations are provided in supplemental tables.

## Results

### Patient characteristics and assessment

Q‐Motor assessments were performed in 46 ataxia patients (ATX; 27 females; age: 50 ± 18 years, range: 12–80), and in 48 sex‐ and age‐matched healthy controls (CON; 33 females, Fisher's exact test: *p* = 0.391; age: 44 ± 18 years, range: 18–88, t‐test: *p* = 0.107). Both groups were predominantly right‐handed (ATX: 40 out of 46, CON: 46 out of 48). Degenerative ataxia was diagnosed in all patients after a full diagnostic work‐up, and a genetic cause was identified in 30 out of 46 patients (see Supplement 1 for list of genotypes and patient characteristics). Overall, ataxia patients covered a broad range of age of onset (38 ± 19 years, range: 1–68), disease duration (12 ± 10 years, range: prodromal to 41), and ataxia severity as captured by the SARA (12 ± 6 points, range: 2–28), FARS‐ADL (12 ± 7 points, range: 1–23), and 9HPT (42 ± 28 s, range: 17–187). Five patients (11%) were no longer able to walk independently (SARA gait item ≥5 points). Median assessment duration was 25 [IQR: 8] minutes (CON: 20 [6] minutes), and data completeness was 98% (CON: 100%), with loss of data for one side during Finger Tapping and Grip‐Lift (*n* = 1); and due to incomplete task execution (*n* = 1), excessive template displacement (*n* = 2), and a software error (*n* = 1) during Spiral Drawing.

### Selection of measures

Overall, 69 out of 188 measures across six movement features and all motor tasks met the selection criteria (Table 1; see Supplement 4 for all selected measures). Movement *speed* and *variability* consistently captured upper limb ataxia in Finger Tapping and diadochokinesia, with moderate‐to‐strong negative correlations of *speed* and positive correlations of *variability* to SARA (|rho_SARA_| = 0.6–0.8), FARS‐ADL (|rho_ADL_| = 0.4–0.7) and 9HPT (|rho_9HPT_| = 0.4–0.6). Reduced limb *stability* during grip‐lift, for example, the measure *position index*, was strongly and positively correlated to ataxia severity (rho_SARA_ = 0.63) and impaired dexterity (rho_9HPT_ = 0.67), but only weakly to functional impairment (rho_ADL_ = 0.35). Tap or grip *force control* consistently failed validation.

**Table 1:** Selection of measures across tasks and movement features.

Measure	Feature	AUC_AvC_	rho_SARA_	rho_SARAul_	rho_ADL_	rho_ADLul_	rho_9HPT_
Finger Tapping							
*Frequency* [ndom]	Speed	**0.89**	**−0.80** ***	**−0.75** ***	**−0.70** ***	**−0.63** ***	**−0.60** ***
STD *T*_*rise*_ [ndom]	Variability	**0.80**	**0.64** ***	**0.58** ***	**0.60** ***	**0.54** ***	**0.58** ***
STD *F*_*AUC*_ [ndom]	Force control	0.76	0.33*	0.36*	0.28	0.27	0.26
Diadochokinesia							
Mean *IPI* [ndom]	Speed	**0.92**	**0.60** ***	**0.62** ***	**0.61** ***	**0.59** ***	**0.57** ***
STD *IPI* [ndom]	Variability	**0.91**	**0.67** ***	**0.60** ***	**0.58** ***	**0.46** ***	**0.46** **
CV *F*_*max*_ [dom]	Force control	0.73	0.54***	0.47***	0.31*	0.35*	0.51***
Grip‐lift							
*Position index* [dom]	Stability	**0.81**	**0.63** ***	**0.62** ***	**0.35** *	**0.34** *	**0.67** ***
*Grip force index* [ndom]	Force control	0.70	0.40**	0.35*	0.15	0.27	0.45**
Spiral Drawing							
*Speed*_*prc90‐100*_	Speed	**0.78**	**0.71** ***	**0.71** ***	**0.32** *	**0.38** *	**0.78** ***
*Distance*_*max*_	Efficiency	0.91	0.57***	0.45***	0.32*	0.30	0.51**
*SPARC*	Smoothness	**0.75**	**−0.71** ***	**−0.75** ***	**−0.43** **	**−0.48** **	**−0.63** ***
*Power*_*1‐4 Hz*_		**0.82**	**0.73** ***	**0.70** ***	**0.38** *	**0.46** **	**0.75** ***
Target Reaching							
*ITI*	Speed	**0.95**	**0.79** ***	**0.76** ***	**0.57** **	**0.59** **	**0.74** ***
*ITI*_*MAD*_	Variability	**0.95**	**0.76** ***	**0.77** ***	**0.56** **	**0.64** ***	**0.73** ***
*Latency*_*acc,2D,MAD*_		**0.85**	**0.82** ***	**0.78** ***	**0.49** **	**0.44** **	**0.78** ***
*Path*_*2D,MAD*_		**0.90**	**0.70** ***	**0.73** ***	**0.51** **	**0.55** **	**0.79** ***
*Path*_*2D*_	Efficiency	**0.90**	**0.70** ***	**0.73** ***	**0.51** **	**0.55** **	**0.79** ***
*Path*_*3D*_		**0.83**	**0.63** ***	**0.71** ***	**0.45** *	**0.52** **	**0.66** ***
*Deviation*_*total*_		**0.93**	**0.63** ***	**0.66** ***	**0.46** *	**0.51** **	**0.65** ***
*SPARC*_*3D*_	Smoothness	**0.80**	**−0.73** ***	**−0.73** ***	**−0.40** *	**−0.49** **	**−0.81** ***
*Hit rate*	Endpoint precision	**0.97**	**−0.81** **	**−0.79** ***	**−0.56** **	**−0.59** **	**−0.80** ***
*Dysmetria*		**0.85**	**0.70** ***	**0.66** ***	**0.45** *	**0.59** **	**0.71** ***

Bold correlations highlight measures that passed selection criteria.

CV, coefficient of variation; dom/ndom, dominant/nondominant hand; *F*_*AUC*_, area under the force curve; IPI, inter‐peak interval; ITI, inter‐tap interval; MAD, median absolute deviation; SPARC, spectral arc length; STD, standard deviation; *T*_*rise*_, duration of tap until peak force.

* *p* < 0.05;

** *p* < 0.01;

*** *p* < 0.001.

Measures of the novel Spiral Drawing and Target Reaching tasks showed the highest correlations to the 9HPT as measure of dexterity (|rho_9HPT_| = 0.63–0.81), and consistently higher correlations to FARS‐ADL upper limb items than to its total score, thus reflecting specific functional impairment in the upper limb domain (Table 1; Supplement 4). Kinematic analysis of reduced *smoothness (SPARC*_*3D*_ *)* captured upper limb ataxia in both tasks and showed particularly strong positive correlations to ataxia severity in the 1–4 Hz frequency band during Spiral Drawing (*Power*_*1‐4Hz*_; rho_SARA_ = 0.73, rho_9HPT_ = 0.75). In Spiral Drawing, *speed* was positively correlated to ataxia severity, with strong correlations of the highest speed decile across a trial (*speed*_*prc90‐100*_; rho_SARA_ = 0.71, rho_ADL_ = 0.32, rho_9HPT_ = 0.78). In Target Reaching, *speed* was negatively correlated to ataxia severity (rho_SARA_ = −0.81, rho_ADL_ = −0.56, rho_9HPT_ = −0.80), and almost perfectly discriminated ataxia patients from controls with a composite of *speed* and *endpoint precision* (*hit rate*, AUC: 0.97). *Efficiency* and spatial *variability* of the reaching movements (*path*_*2D*_ and *path*_*2D,MAD*_), as well as *variability* in the timing of maximum acceleration (*latency*_*acc,2D,MAD*_) best captured upper limb ataxia based on the virtual 2D projection, not the actual 3D trajectory of the movement (Table 1).

While healthy controls were age‐ and sex‐matched to validate discrimination from ataxia patients, a separate analysis of the control cohort showed that age is a potential covariate in 34 out of 69 selected measures. Moreover, 46/69 measures correlated to dexterity even though variability of the 9HPT was small among controls (20 ± 4 s; see Supplement 5 for analysis of healthy controls).

### Discrimination between severity levels

**Figure 3:**
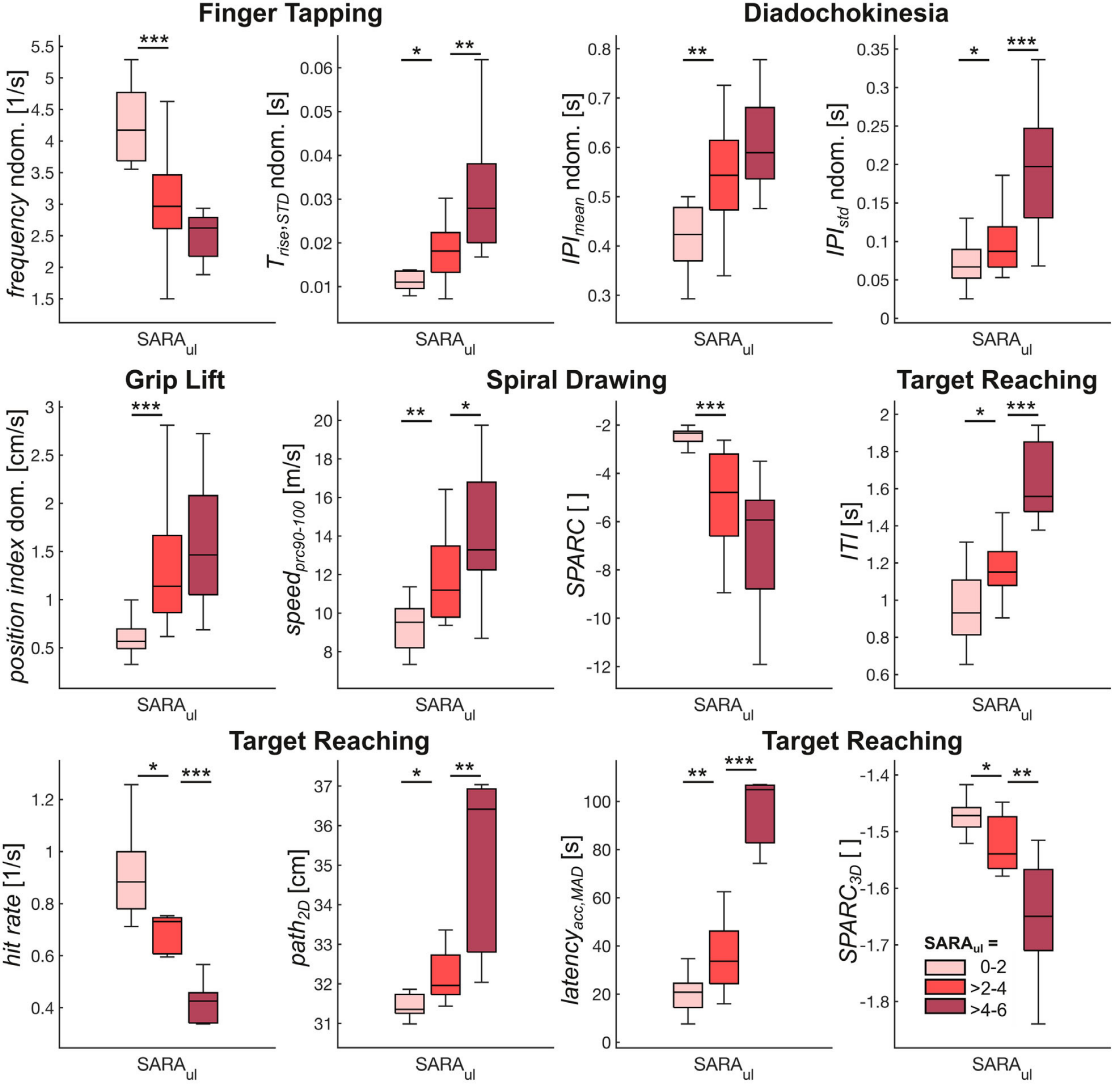
Representative measures across all tasks and features with significant discrimination between three severity levels of upper limb ataxia, as quantified by the composite of upper limb items of the Scale for the Assessment and Rating of Ataxia (SARA_ul_). Asterisks indicate significant differences (*p < 0.05, **p < 0.01, and ***p < 0.001).

To examine and compare the discriminatory power of the selected measures between different severity levels of upper limb ataxia, the cohort was split into three groups of mild (SARA_ul_: 0–2, *n* = 15), moderate (>2–4, *n* = 17), and severe (>4–6, *n* = 14) upper limb ataxia. All 69 selected measures discriminated between the three severity levels (Kruskal–Wallis test, all *p* < 0.018), and measures in all corresponding movement features and across all tasks discriminated between mild and moderate upper limb ataxia in their order of severity (Wilcoxon rank‐sum test, *p* ≤ 0.046 for all respective measures; Fig. 3; see Supplement 6 for all measures and differences between severity levels). Patients with moderate and severe upper limb ataxia were best discriminated by movement *variability—*but not *speed—*of Finger Tapping and diadochokinesia, and all features of Target Reaching (*p* ≤ 0.009 for respective measures).

### Sensitivity to mild ataxia

Because future trials will likely target early disease stages, we characterized the particular sensitivity of quantitative motor assessment of upper limb function in a sub‐cohort of mild ataxia patients (SARA ≤10; *n* = 20). Compared to the full cohort, variability of data in this sub‐cohort was smaller for the SARA (7 ± 3 points, range: 2–10; SARAul: 0–3.5), FARS‐ADL (8 ± 5 points, range: 1–15), and the 9HPT (29 ± 8 s, range: 17–53). Nevertheless, *speed* of drawing (*acc*_*prc90*_), reduced *smoothness* of drawing (*acc*_*MAD*_), and spatial *variability* in the 2D reaching trajectory (*path*_*2D,MAD*_) all discriminated ataxia patients from healthy controls (AUC = 0.75–0.85) and had moderate‐to strong positive correlations to each the SARA (rho_SARA_ = 0.54–0.62), the FARS‐ADL (rho_ADL_ = 0.47–0.54) and the 9HPT (rho_9HPT_ = 0.67–0.76), thus showing that the novel Spiral Drawing and Target Reaching tasks can capture upper limb ataxia even in mild ataxia patients. Additional 16 measures showed strong, yet complementary correlations to one or two of these COAs, including highest correlations to impaired function in the FARS‐ADL (*speed* of Finger Tapping and diadochokinesia) or impaired dexterity in the 9HPT (*variability* and *efficiency* of Target Reaching; Table 2; see Supplement 7 for all measures).

**Table 2:** Selection of measures in mild ataxia (SARA≤10).

Measure	Feature	AUC_AvC_	rho_SARA_	rho_SARAul_	rho_ADL_	rho_ADLul_	rho_9HPT_
Finger Tapping							
Mean *IPI* [ndom]	Speed	0.79	0.48*	0.49*	0.61**	0.34	0.44
Diadochokinesia							
*Frequency* [ndom]	Speed	0.87	−0.30	−0.28	−0.62**	−0.41	−0.26
Spiral Drawing							
*acc*_*prc90*_	Speed	**0.75**	**0.61** **	**0.37**	**0.47** *	**0.45** *	**0.67** **
*acc*_*MAD*_	Smoothness	**0.75**	**0.62** **	**0.37**	**0.47** *	**0.46** *	**0.69** **
Target Reaching							
*path*_*2D,MAD*_	Variability	**0.85**	**0.54** *	**0.37**	**0.54** *	**0.45**	**0.76** **
*dtw*_*3D*_		0.81	0.32	0.26	−0.07	−0.03	0.75**
*path*_*2D*_	Efficiency	0.90	0.50*	0.52	0.33	0.44	0.73**

Bold correlations highlight measures that passed selection criteria.

acc, acceleration; dom/ndom, dominant/nondominant hand; dtw, dynamic time warp; IPI, inter‐peak interval; MAD, median absolute deviation.

* *p* < 0.05;

** *p* < 0.01.

## Discussion

To develop digital‐motor outcomes for upper limb ataxia, this exploratory study extended quantitative motor assessment with the trial‐ready Q‐Motor battery by two novel Spiral Drawing and Target Reaching tasks, and performed a systematic validation in a large cross‐sectional ataxia cohort. Overall, we demonstrated that this extended quantitative motor assessment comprehensively captures upper limb ataxia across multiple features in all motor tasks, and with measures that reflect clinician‐reported (SARA), patient‐focused (FARS‐ADL), and performance outcomes (9HPT) as key clinical outcome domains of ataxia.

For the established Finger Tapping, diadochokinesia, and grip‐lift tasks, this validation is consistent with—and extends—a previous study in FA,^39^ now demonstrating how digital‐motor measures reflect upper limb ataxia across a broader spectrum of degenerative ataxias, and within a systematic framework of overarching movement features. As key feature, *speed* was negatively and consistently correlated with ataxia severity across all established tasks. This underlines that *speed* is a central and measurable error trade off when voluntary movements are impaired by ataxia.^26^ Increased movement *variability* not only captured upper limb ataxia during speeded diadochokinesia as a gross motor task (as expected from bedside tests), but equally well during speeded index Finger Tapping as a simple distal fine motor task. Notably, upper limb ataxia was best captured in both tasks by the *variability* of subsegments of the tap defined by force dynamics such as the duration until peak force (*T*_*rise*_). This assessment of tapping dynamics relied on the precise force sensor of our setup. While cross‐validation with remote assessments will be required in the future, this precise assessment of tapping may not be fully achieved in task implementations on smartphones/tablets, in which finger surface areas on the display provide only indirect measures of force, or in clicking tasks based on computer mouse or keyboard with binary input and variable mechanic and kinematic properties of the device.^12^ Reduced limb *stability* during grip‐lift possibly reflected cerebellar postural tremor, given that patients with significant non‐cerebellar movement disorders (e.g., myoclonus) had been excluded.

To achieve a more comprehensive assessment of upper limb ataxia, the present study implemented and validated Spiral Drawing and Target Reaching as two novel tasks targeting kinematic impairment as key deficit in ataxia. Consistent with their rationale and previous kinematic studies of upper limb movement in ataxia,^12^, ^13^, ^26^, ^37^, ^40^ these tasks best captured upper limb functional impairment across all motor tasks, particularly with the kinematic analysis of *smoothness*. Moderate‐to‐strong correlations to upper limb item composites of SARA and FARS ADL—higher than to their total scores—suggests specificity of the respective measures for functional deficits in the upper limb domain, rather than an unspecific association with a general underlying construct of ataxia. In addition, measures of reduced *smoothness* showed the overall highest positive correlation to the 9HPT, thus best reflecting impaired dexterity in ataxia. In Spiral Drawing, the quantification of *smoothness* was particularly sensitive to appendicular ataxia in the frequency band of 1–4 Hz. This range appears to be consistent with the frequency of cerebellar intention tremor, although the phenomenology of cerebellar tremors is broad, and task‐specific tremors and frequencies are insufficiently characterized in degenerative ataxias.^41^ The *speed* of Spiral Drawing in ataxia patients was skewed toward higher speeds, at least if calculated as spatial displacement (cm/s). This spatial displacement presents a measure of increased cumulative movement in all unwanted directions relative to the template, and is not inconsistent with decreased angular velocity (degree/s) as a measure of effective drawing speed along the template.^30^ In Target Reaching, *hit rate*—a composite of *endpoint precision* and *speed*‐ almost perfectly discriminated ataxia patients from controls. This metric performance depended critically on the sequential 4‐target design. A preliminary 2‐target left–right pointing task, similar to the analogue “click task” with counting of target hits per second,^42^ was aborted due to poor correlations with ataxia severity. For the *efficiency* and *variability* of reaching movements, our study is the first to demonstrate that the severity of upper limb ataxia may be better captured in the planar 2D projection than in the actual 3D coordinates of the movement (Table 1). Specifically, we show that the respective measures for the movement in the 2D plane can be sufficiently extracted virtually, while kinematic studies typically control for planar movements by physical restrictions.^37^ Movement in the vertical axis was probably subject to larger behavioral variability because task requirements in this dimension were less defined. Alternatively, vertical movement might have required less cerebellar control due to exploitation of gravity,^43^ or its relatively short lifting trajectory that required only a single‐joint shoulder movement as compared to the multi‐joint movement and torque interactions during reaching in the horizontal plane.^44^

As key requirement for implementation in ataxia clinical trials, digital motor outcomes must be sensitive to changes of disease severity (due to progression or treatment), and they should be able to capture disease severity in the most trial‐relevant early ataxia stage. This study demonstrated that measures in all tasks can discriminate between three severity levels of upper limb ataxia, particularly between mild and moderate severity (as defined by the composite of SARA upper limb items: 0–2 vs. >2–4). Without external anchors for the grading of upper limb ataxia, our binning of severity levels based on the SARA was arbitrary, but nevertheless showed that discrimination by quantitative motor assessment is possible even within a narrow range of only 4 out of 12 possible SARA points that span ataxia severity in the upper limb domain. In a subgroup of patients with mild ataxia (defined by SARA<10)^2^, we showed that Spiral Drawing and Target Reaching capture upper limb ataxia even in early disease stages. The amendment of these two novel tasks was thus not only informed by theoretical task design and rationale, but is also most likely to yield sensitive outcome measures in relevant trial scenarios. For both tasks, the kinematic analysis of measures such as *speed*, *acceleration*, or *smoothness* may be implementable as remote assessment in the future, as shown for drawing and reaching on tablets/computers,^12^, ^30^, ^45^ or the assessment of clinical tests^13^, ^26^ and instrumented tasks^9^ with wearable accelerometers. In contrast, our high‐resolution spatial measures of Target Reaching (i.e., path *efficiency* and *variability*), which were particularly informative in mild ataxia, cannot be accurately analyzed with accelerometers. Here, however, our laboratory‐based quantitative motor assessment provides a distinct and novel advantage over experimental camera‐ and reflector‐based tracking systems unsuitable for clinical trial settings.^37^, ^40^

While this exploratory study provides a fist validation in a large cohort of ataxia patients and against key clinical outcome domains, it is limited by the representation of the cohort and its cross‐sectional character. Study inclusion was conceptually open to nonambulatory patients, in whom digital‐motor assessment of upper limb ataxia would be particularly important for future trial scenarios (because gait and stance can no longer be assessed). However, the actual number of patients who were no longer able to walk independently was unexpectedly low, probably because of bias toward less advanced ataxia patients being able to present to clinic, and/or the exclusion of patients with functionally relevant non‐cerebellar impairment, which is common in most advanced genetic ataxias.^2^ Further, this study aimed to validate the quantitative motor assessment of upper limb movements generically across many degenerative ataxias, including both relatively common and ultra‐rare genetic ataxias, for which separate validation will never be possible. Although phenotypical assessments of the motor domain may well be transferable across ataxias, additional validation studies in genetically stratified cohorts are thus still warranted before implementation as trial outcome. This may also show that optimal digital‐motor tasks and measures vary between ataxia genotypes. For example, the severity of ataxias with prominent oculomotor impairment (e.g., SCA2), which was not controlled for in this study, might be particularly captured by visually directed Target Reaching, independent of upper limb ataxia. Finally, measures of this study remain to be validated in longitudinal studies to determine their sensitivity to the change in trial‐relevant time windows, but also their test–retest reliability in shorter periods. Such validation will further shorten the list of potential digital motor measures, and eventually benefit from multivariate analyses to explore the interdependency of measures^38^ or the added value of composites.^9^, ^26^ In Huntington's disease, being a similarly genetically defined progressive neurodegenerative disease covering the motor domain, the high cross‐sectional validity of individual Q‐Motor measures was subsequently confirmed in longitudinal analyses in the large biomarker study TRACK‐HD.^46^, ^47^ For ataxias, chances should be high to observe similar behavior, and longitudinal validation is ongoing, together with additional analyses of patient meaningfulness. If ultimately reliable and responsive to disease progression, its proof‐of‐principle regulatory acknowledgment as clinical endpoint in trials and its availability at more than 150 sites worldwide could make Q‐Motor rapidly applicable in upcoming ataxia clinical trials.

## Author Contributions

All authors contributed to the conception and design of the study. D.H., Ro.S., P.B., and A.T. contributed to the acquisition, analysis, or interpretation of data. D.H. and A.T. contributed to drafting the text and preparing the figures. All authors critically reviewed the manuscript for important intellectual content.

## Conflict of Interest

Ro.S. and P.B. are employees, and R.R. is director of both the George‐Huntington‐Institute and QuantiMedis manifacturing Q‐Motor devices and Software and providing Q‐Motor services for research projects and sponsors. M.S. has received consultancy honoraria from Ionis, UCB, Prevail, Orphazyme, Servier, Reata, GenOrph, AviadoBio, Biohaven, Zevra, and Lilly, all unrelated to the present manuscript. All other authors declare that they have no conflict of interest.

## Supporting information


Supplement 1.

## Data Availability

Anonymized data will be made available upon reasonable request.
