# Supplementary material for: Multifeature quantitative motor assessment of upper limb ataxia including drawing and reaching
Source: Ann Clin Transl Neurol. 2024 Apr 8;11(5):1097–109. doi: 10.1002/acn3.52024 (PMC11093241; doi:10.1002/acn3.52024)
Supplement: Supplementary file 1 — Supplement 1. [file ACN3-11-1097-s001.docx]

**Supplement 1: Detailed patient characteristics**

| ID | Clinical or genetic diagnosis** | Sex | Age  [yr] | Onset  [yr] | Duration  [yr] | SARA score | FARS ADL | 9HPT dom [s] |
| --- | --- | --- | --- | --- | --- | --- | --- | --- |
| ATX01 | SCA28 | F | 66 | 38 | 28 | 14 | 23 | 37 |
| ATX02 | PNPLA6 ataxia | F | 48 | 38 | 10 | 19 | 17 | 34 |
| ATX03 | SCA2 | F | 38 | 28 | 10 | 22 | 21 | 187 |
| ATX04* | SCA7 | M | 44 | 38 | 6 | 8 | 14 | 53 |
| ATX05* | SCA14 (VUS) | M | 71 | 30 | 41 | 10 | 6 | 41 |
| ATX06* | Congenital ataxia with cerebellar ataxia, mild spasticity, myoclonus, and neuropathy (unsolved) | F | 29 | 1 | 28 | 10 | 13 | 37 |
| ATX07 | ILOCA | F | 54 | 49 | 5 | 16 | 16 | 79 |
| ATX08 | Congenital ataxia with cerebellar ataxia, mild retardation, neuropathy, and myoclonus (unsolved) | M | 18 | 1 | 17 | 13 | 5 | 41 |
| ATX09 | SCA2 | F | 30 | 20 | 10 | 13 | 6 | 38 |
| ATX10* | POLG ataxia | F | 29 | 22 | 7 | 4 | 2 | 20 |
| ATX11 | FXTAS | F | 80 | 65 | 15 | 18 | 19 | 76 |
| ATX12 | ADCA (unsolved) | F | 78 | 63 | 15 | 10 | 11 | 29 |
| ATX13* | SCA19 | F | 60 | 25 | 35 | 9 | 8 | 26 |
| ATX14 | Sensory and cerebellar ataxia (unsolved) | M | 63 | 53 | 10 | 14 | 17 | 36 |
| ATX15 | SCA1 | F | 32 | 27 | 5 | 14 | 20 | 45 |
| ATX16* | Episodic Ataxia Type 2 | F | 48 | 10 | 38 | 8 | 10 | 22 |
| ATX17 | SCA1 | M | 31 | 24 | 7 | 18 | 14 | 72 |
| ATX18 | SCA15 | F | 61 | 41 | 20 | 13 | 14 | 42 |
| ATX19 | SCA15 | M | 35 | 30 | 5 | 17 | 17 | 109 |
| ATX20 | ILOCA | M | 57 | 49 | 8 | 13 | 11 | 37 |
| ATX21 | ADCA (unsolved) | M | 62 | 58 | 4 | 11 | 19 | 39 |
| ATX22* | ADCA (unsolved) | F | 71 | 60 | 11 | 6 | 15 | 26 |
| ATX23 | ILOCA | M | 61 | 51 | 10 | 11 | 10 | 34 |
| ATX24* | Early-onset ataxia (unsolved) | F | 53 | 38 | 15 | 7 | 8 | 21 |
| ATX25 | POLG ataxia | F | 44 | 41 | 3 | 12 | 16 | 33 |
| ATX26* | Episodic Ataxia Type 2 | M | 72 | 64 | 8 | 2 | 5 | 24 |
| ATX27* | SCA6 (prodromal) | F | 57 | 57 | 0 | 3 | 1 | 17 |
| ATX28 | AOA2 | M | 60 | 40 | 20 | 15 | 17 | 47 |
| ATX29 | Early-onset cerebellar ataxia with mental retardation and pyramidal signs (unsolved) | F | 56 | 32 | 24 | 12 | 23 | 25 |
| ATX30 | SCA48 | M | 36 | 30 | 6 | 18 | 11 | 61 |
| ATX31* | Mixed sensory and cerebellar ataxia with  mild cognitive impairment (unsolved) | M | 69 | 62 | 7 | 7 | 5 | 29 |
| ATX32* | ILOCA | M | 78 | 68 | 10 | 7 | 3 | 27 |
| ATX33* | Cerebellar ataxia with dysautonomia (unsolved) | F | 70 | 57 | 13 | 10 | 21 | 27 |
| ATX34 | Niemann-Pick Type C | F | 34 | 15 | 19 | 12 | 11 | 41 |
| ATX35* | SCA23 | M | 72 | 65 | 7 | 7 | 3 | 28 |
| ATX36* | SCA5 | F | 49 | 38 | 11 | 4 | 7 | 22 |
| ATX37* | ADCA (unsolved) | F | 51 | 49 | 2 | 9 | 17 | 31 |
| ATX38* | ANO10 ataxia | M | 56 | 48 | 8 | 10 | 10 | 35 |
| ATX39 | Multiple System Atrophy, cerebellar type | F | 62 | 60 | 2 | 17 | 23 | 33 |
| ATX40* | SCA48 | M | 49 | 47 | 2 | 8 | 6 | 31 |
| ATX41 | Friedreich Ataxia (late-onset) | F | 37 | 32 | 5 | 15 | - | 27 |
| ATX42 | Friedreich Ataxia (late-onset) | F | 50 | 42 | 8 | 13 | - | 44 |
| ATX43 | Friedreich Ataxia | M | 12 | 8 | 4 | 16 | 10 | 43 |
| ATX44 | Friedreich Ataxia | M | 20 | 12 | 8 | 28 | 20 | 52 |
| ATX45 | Friedreich Ataxia | F | 21 | 12 | 9 | 26 | 19 | 66 |
| ATX48* | Episodic Ataxia Type 2 | F | 39 | 12 | 27 | 3 | 5 | 24 |

* = subcohort with ‘mild ataxia’ (SARA≤10); ** degenerative cerebellar ataxias with or without sensory ataxia (e.g. including FA), and non-cerebellar features not severe enough to cause additional relevant impairment; 9HPT = 9-Hole Peg-Test; ADCA = Autosomal-dominant cerebellar ataxia; ADL = Activities of daily living; dom = dominant hand; ILOCA = Idiopathic late-onset cerebellar ataxia

**Supplement 2: Detailed description of methods**

*Setup*

Depending on the Q-Motor task, measurements were based on a customized pre-calibrated force-transducer to capture tap and grip forces at a sampling frequency of 350 Hz, and/or an electromagnetic position sensor (Polhemus Inc., Colchester, VT) capturing the position and angle of hand-held objects (during lifting, drawing, and reaching) at a sampling frequency of 120 Hz, a spatial resolution of <0.076 mm, and an angular resolution of <0.0147°. Subjects were seated in a standard chair of fixed height centered in front of the setup, and non-ambulatory subjects were transferred from their wheelchair to the standard chair for equal measurement conditions. Acquired raw data were transferred to the George Huntington Institute for central quality control and analysis using automated algorithms, blinded to clinical characteristics of the ataxia patients.

*Finger Tapping (Digitomotography) and Diadochokinesia (Dysdiadochomotography)*

Speeded Finger Tapping (Digitomotography) required repeated tapping on the force sensor with the index finger as fast as possible for 10 seconds. Similarly, speeded Diadochokinesia assessment (Dysdiadochomotography) required alternating pronation and supination tapping with the distal palm and back of the hand, as fast as possible for 10 seconds. The beginning and end of each trial were signaled by an audio cue. Based on the respective time-force series during Finger Tapping and Diadochokinesia, extracted measures included the tapping *frequency* [taps per second], and, for each tap interval or tap, the *inter-onset interval (IOI)* [s], *inter-tap interval (ITI)* [s], *inter-peak interval (IPI)* [s], *tap duration (TD)* [s], and *maximum tap force (F_max_)* [N], as previously described[1, 5]. Novel measures comprised the separation of the tap duration into the *rising edge (T_rise_)* and *falling edge (T_fall_)* before and after reaching the maximum tap force, and the *area under the curve (F_AUC_)* of the tap force over the full tap duration. All tapping measures were log-transformed, and their mean, standard deviation (STD), and coefficient of variation (CV; STD/mean) were calculated based on their *within-trial* variability across taps. All measures were then averaged across the three repeated trials in the dominant and non-dominant hand, respectively. Intraclass correlation coefficients (ICC) between the first and the third trial are provided for each measure in Supplemental 4.

*Grip Lift (Manumotography & Hyperkinesiomotography)*

A Grip-Lift task required lifting and holding a 250 g object in a precision grip of thumb and index finger with the unsupported arm, as stable as possible for 20 seconds. The beginning and end of each trial were signaled by an audio cue. Measures of involuntary movement (Hyperkinesiomotography) and precision grip force (Manumotography) were calculated for the static holding phase between 10 seconds from trial onset to the end of the 20 second trial[3-5]. From the time-series of the position and angle of the object, the *position index (PI)* and *orientation index (OI)* were calculated as the Euclidean norm of Cartesian x/y/z positions and yaw/pitch/roll angles, respectively. The time-series of the force sensor below the thumb was used to calculate the kinetic measures *mean grip force (F_mean_)* [N] and *grip force variability (F_cv_)* as given by its coefficient of variation within a trial, and the *grip force index (FI)* as the Euclidean norm of forces along 3 axes (orthogonal and in plane to sensor surface). All measures were then averaged across the three repeated trials in the dominant and non-dominant hand, respectively. Intraclass correlation coefficients (ICC) between the first and the third trial are provided for each measure in Supplemental 4.

*Spiral Drawing*

Subjects were required to trace a 5 cm diameter Archimedes spiral on a paper template from the inside out using a digitizer pen (Polhemus FASTRAK, Polhemus Inc., Colchester, VT) with an attached pencil lead. With their forearms resting on the table, subjects were instructed to trace the template ‘as accurately as possible’ (i.e. irrespective of duration) and ‘as smoothly as possible’ (i.e. without intermittent lift-offs and pauses). The beginning of a trial was signaled with an audio cue after the subject had securely placed the digitizer pen in the center of the spiral template, and stopped manually when the subject reached the outer end of the template. Each subject performed two trials (practice trial, and test trial used for analysis) with their dominant hand, after familiarization with the digitizer pen by writing their name. Preprocessing of the raw 3D positional data comprised removal of all time bins before and/or after tracing the template, and during unexpected vertical lift-offs more than 3 mm from the board level. After this process, the vertical dimension was discarded from the data, yielding the 2D positional data of the digitizer pen parallel to the drawing plane.

To extract spatial measures, the 2D positional data was transformed into polar coordinates and fitted to a digital copy of the paper template. The digital template was sampled at 1000 angular phases (corresponding to an angular resolution of 1.8° for five windings of 360°), and each of these points on the template was matched to the corresponding point on the subjects´ trace with minimum difference in angular phase. The Euclidean distance between all matched points was used to calculate their cumulative sum *(distance_total_)*, their robust median *(distance_MED_)* and median absolute deviation *(distance_MAD_)*, their maximum distance from the template *(distance_max_)*, their 90th percentile *(distance_prc90_)*, and the cumulative deviation across the highest decile *(distance_prc90-100_)*. To account for behavioral variability and errors, the first 360° winding was discarded from the analysis of spatial measures, and trials with less than five 360° spiral traces due to ‘shortcuts’ between windings were excluded. To account for potential paper displacement relative to the measurement frame, the analysis of spatial measures was performed for the optimal fit between patient trace and digital template. This optimal fit was obtained by minimization of *distance_total_* for spatial shifts of the spiral center within ± 15 mm in the 2D plane.

Measures in the spatiotemporal and frequency domain were calculated based on the first (i.e. *speed*) and second (i.e. acceleration, *acc*) temporal derivative of the pre-processed 2D positional data, each followed by digital filtering with a 4th order 8 Hz Butterworth filter. Based on the distribution of instantaneous speeds and (absolute values of) accelerations across the trial duration, we calculated their respective median, median absolute deviation, 90th percentile, and cumulative total across the highest decile (e.g. *speed_MED_, speed_MAD_, speed_prc90_, speed_prc90-100_*) as spatiotemporal measures, given that speed and acceleration appeared to be skewed towards higher values in ataxia patients (Fig 1C). Fast Fourier transform analysis (FFT) was applied to calculate smoothness of movement by means of the Spectral Arc Length *(SPARC)* of the power spectrum of the time-speed series, based on open-source algorithms[2]. In addition, manual comparison of the power spectrum between ataxia patients and healthy controls revealed a consistent peak of power in ataxia patients between 1 and 4 Hz (Fig 1D). Thus, the cumulative power in this narrow frequency band (Power_1-4Hz_) was calculated as an additional smoothness measure in the frequency domain.

*Target Reaching*

Subjects were required to perform an ordered sequence of multi-joint reaching and pointing movements[6]. With a hand-held digitizer stylus (FASTRAK Digitizer, Polhemus Inc., Colchester, VT), subjects were instructed to point -‘as fast and accurately as possible’- alternately between four red circular targets of 1.5 cm diameter: a starting target S in front of them, and one of three targets 25 cm away in the anterior-posterior dimension (see Fig. 1A and 2A,C,E for illustration). The center target C was in midline with S, and targets R and L 20 cm right and left from the midline. The task was performed with the dominant hand, unsupported by elbow or forearm. With an audio cue, pointing started with a reaching movement towards the target ipsilateral to the dominant hand, and continued in serial reaching and pointing sequences from the ipsilateral to the center to the contralateral target. Thus, the target sequence was S-R-S-C-S-L-S-R-S-… for right-handed subjects, and S-L-S-C-S-R-S-L-S-…for left-handed subjects. The trial was stopped manually after the completion of 10 reaching movements per target and direction. Each subject performed two trials (practice trial, and test trial used for analysis) without prior knowledge of the required number of repetitions. The extraction of measures is fully explained in the main text of the manuscript.

**References**

1. Bechtel N, Scahill R, Rosas HD, Acharya T, van den Bogaard SJ, Jauffret C, Say MJ, Sturrock A, Johnson H, Onorato CE (2010) Tapping linked to function and structure in premanifest and symptomatic Huntington disease. Neurology 75:2150-2160

2. Mohamed Refai MI, Saes M, Scheltinga BL, van Kordelaar J, Bussmann JB, Veltink PH, Buurke JH, Meskers CG, van Wegen EE, Kwakkel G (2021) Smoothness metrics for reaching performance after stroke. Part 1: which one to choose? Journal of neuroengineering and rehabilitation 18:1-16

3. Reilmann R, Bohlen S, Kirsten F, Ringelstein EB, Lange HW (2011) Assessment of involuntary choreatic movements in Huntington's disease—toward objective and quantitative measures. Movement disorders 26:2267-2273

4. Reilmann R, Bohlen S, Klopstock T, Bender A, Weindl A, Saemann P, Auer DP, Ringelstein EB, Lange HW (2010) Grasping premanifest Huntington's disease–shaping new endpoints for new trials. Movement disorders 25:2858-2862

5. Reilmann R, Schubert R (2017) Motor outcome measures in Huntington disease clinical trials. Handb Clin Neurol 144:209-225

6. Sanguineti V, Morasso PG, Baratto L, Brichetto G, Mancardi GL, Solaro C (2003) Cerebellar ataxia: Quantitative assessment and cybernetic interpretation. Human Movement Science 22:189-205

**Supplement 3: Matrix of measures across movement features and motor tasks**

|  | Finger Tapping/ Diadochokinesia | Grip-Lift | Spiral Drawing | Target Reaching |
| --- | --- | --- | --- | --- |
| Speed | *frequency*  Mean of *IOI, IPI, ITI, TD, T_rise_, T_fall_* | - | *speed_MED_, speed_max_,  speed_prc90_, speed_prc90-100_*  *acc_MED_, acc_max_,  acc_prc90_, acc_prc90-100_* | *frequency*  *ITI, TD*  *speed_mean_, acc_mean_, dec_mean_* in 3D/2D  *speed_max_, acc_max_, dec_max_* in 3D/2D  *latency_speed_, latency_acc_, latency_dec_* in 3D/2D |
| Variability | CV of *IOI, IPI, ITI, TD, T_rise_, T_fall_*  STD* of *IOI, IPI, ITI, TD, T_rise_, T_fall_* | - | - | *ITI_MAD_ and TD_MAD_*  *speed_mean,MAD_, acc_mean,MAD_, dec_mean,MAD_* in 3D/2D  *speed_max,MAD_, acc_max,MAD_, dec_max,MAD_* in 3D/2D  *latency_speed,MAD_, latency_acc,MAD_, latency_dec,MAD_* in 3D/2D  *path_3D,MAD_, path_2D,MAD_, path_AP,MAD_, path_LR,MAD_, path_V,MAD_*  *dtw* in 3D/2D |
| Efficiency | - | - | *distance_MED_, distance_MAD_, distance_prc90_, distance_prc90-100_, distance_total_, distance_max_* | *path_3D_, path_2D_, path_ap_, path_lr_, and path_v_*  *deviation_max_*  *deviation_total_**** |
| Smoothness | - | - | *speed_MAD_, acc_MAD_*  *SPARC*  *Power_1-4 Hz_* | *SPARC* in 3D/2D |
| Endpoint precision | - | - | - | *distance*  *dysmetria*  *hit rate***** |
| Stability | - | *position index (PI)*  *orientation index (OI)* | - | - |
| force control | Mean, STD*, and CV of *F_max_*  Mean, STD*, and CV of *AUC*** | *F_mean_, F_cv_*  *grip force index (FI)* | - | - |
| TOTAL (n=188) | 2 x 25 = 50 measures  (dom/ndom) | 5 measures (dom/ndom) | 18 measures  (dom) | 60 measures  (dom) |

3D/2D = 3D trajectory and virtual projection on 2D plane; AUC = area under the curve; CV = coefficient of variation; dom/ndom = dominant/non-dominant hand;
DTW = dynamic time warp; IOI = inter-onset interval; IPI = inter-peak interval; ITI = inter-tap interval; MAD = median absolute deviation; MED = median; STD = standard deviation; SPARC = Spectral Arc Length; TD = tap duration; T_rise_ = duration of tap until peak force; T_fall_ = duration of tap after peak force.

* STD representing composite of speed and variability (STD = MEAN x CV, given that STD ~ MEAN)
** AUC representing composite of speed and grip force control (area under the time-force curve of a tap)
*** Deviation_total_ representing composite of speed and efficiency (cumulative tremor amplitude across movement duration)
**** Hit rate representing composite of speed and endpoint precision (number of target hits per second)

**Rationale**

Measures were grouped into seven physiologically interpretable movement features to demonstrate how they systematically capture different phenomenological and functional aspects of upper limb ataxia across these feature dimensions. To this end, we adopted and combined features from previous studies using pure kinematic data[1] or joint kinematic and kinetic assessment[2], and delineated subcomponents to map the comprehensive parameter space of our extended assessment. Within the recently suggested STAR domains, our movement features *smoothness* and *stability* would both be classified as Stability (“unnecessary movements in secondary axes”). Here, we separate this dimension into stability of movement (kinematic *smoothness*) and stability of position (stationary holding of object, i.e. positional *stability*). Movement *speed* would be classified as Timing (with speed as error trade off), *efficiency* and *endpoint precision* both as Accuracy (spatial errors), and variability as Rhythmicity (irregularity in repeated movements). Variability was more broadly defined in this study, covering spatial (e.g. path length), temporal (e.g. inter-tap interval), spatiotemporal (e.g. latency to maximum speed), and also structural aspects (dynamic time warp for the variability of the full time-speed series). Together, these features presented an a priori hypothesis-driven framework for cerebellar impairment of voluntary upper limb movements, which handles redundancy and enables functional interpretation of a complex parameter space in the absence of a clearly defined construct of ‘appendicular ataxia’.

**References**

1. Bipasha K, Dung P, Pathirana PN, Horne M, Power L, Szmulewicz D (2020) Objective Assessment of Cerebellar Ataxia: A Comprehensive and Refined Approach. Scientific Reports (Nature Publisher Group) 10

2. Kanzler CM, Lessard I, Gassert R, Brais B, Gagnon C, Lambercy O (2023) Digital health metrics reveal upper limb impairment profiles in ARSACS. Journal of the Neurological Sciences:120621

**Supplement 4: Complete list of selected measures across tasks and movement features.**

| **Measure** | **Feature** | **AUC_AvC_** | **rho_SARA_** | **rho_SARAul_** | **rho_ADL_** | **rho_ADLul_** | **rho_9HPT_** | **ICC** |
| --- | --- | --- | --- | --- | --- | --- | --- | --- |
| **Finger Tapping** |  |  |  |  |  |  |  |  |
| Mean *TD* [dom] | Speed | 0.83 | 0.67*** | 0.59*** | 0.53*** | 0.50*** | 0.56*** | 0.98 |
| Mean *TD* [ndom] | Speed | 0.84 | 0.70*** | 0.67*** | 0.65*** | 0.57*** | 0.52*** | 0.96 |
| Mean *T_fall_* [dom] | Speed | 0.87 | 0.68*** | 0.55*** | 0.56*** | 0.42** | 0.59*** | 0.95 |
| Mean *T_fall_* [ndom] | Speed | 0.85 | 0.72*** | 0.67*** | 0.64*** | 0.52*** | 0.59*** | 0.93 |
| *frequency* [dom] | Speed | 0.91 | -0.80*** | -0.70*** | -0.67*** | -0.56*** | -0.62*** | 0.96 |
| *frequency* [ndom] | Speed | 0.89 | -0.80*** | -0.75*** | -0.70*** | -0.63*** | -0.60*** | 0.97 |
| Mean *IOI* [dom] | Speed | 0.91 | 0.80*** | 0.70*** | 0.66*** | 0.55*** | 0.62*** | 0.97 |
| Mean *IOI* [ndom] | Speed | 0.89 | 0.80*** | 0.75*** | 0.70*** | 0.63*** | 0.59*** | 0.97 |
| Mean *IPI* [dom] | Speed | 0.91 | 0.73*** | 0.61*** | 0.65*** | 0.44** | 0.52*** | 0.96 |
| Mean *IPI* [ndom] | Speed | 0.89 | 0.79*** | 0.74*** | 0.73*** | 0.62*** | 0.57*** | 0.96 |
| Mean *ITI* [dom] | Speed | 0.89 | 0.67*** | 0.56*** | 0.56*** | 0.43** | 0.53*** | 0.96 |
| Mean *ITI* [ndom] | Speed | 0.88 | 0.78*** | 0.73*** | 0.64*** | 0.59*** | 0.63*** | 0.96 |
| STD *T_rise_* [ndom] | Variability | 0.80 | 0.64*** | 0.58*** | 0.60*** | 0.54*** | 0.58*** | 0.73 |
| **Diadochokinesia** |  |  |  |  |  |  |  |  |
| *frequency* [ndom] | Speed | 0.92 | -0.57*** | -0.59*** | -0.63*** | -0.60*** | -0.52*** | 0.93 |
| Mean *IOI* [ndom] | Speed | 0.92 | 0.58*** | 0.59*** | 0.63*** | 0.60*** | 0.52*** | 0.91 |
| Mean *IPI* [ndom] | Speed | 0.92 | 0.60*** | 0.62*** | 0.61*** | 0.59*** | 0.57*** | 0.92 |
| Mean *ITI* [ndom] | Speed | 0.89 | 0.58*** | 0.60*** | 0.42** | 0.44** | 0.58*** | 0.91 |
| STD *T_fall_* [ndom] | Variability | 0.88 | 0.60*** | 0.53*** | 0.60*** | 0.51*** | 0.48** | 0.78 |
| STD *IOI* [ndom] | Variability | 0.88 | 0.62*** | 0.55*** | 0.52*** | 0.41** | 0.42** | 0.86 |
| STD *IPI* [ndom] | Variability | 0.91 | 0.67*** | 0.60*** | 0.58*** | 0.46** | 0.46** | 0.79 |
| STD *ITI* [ndom] | Variability | 0.88 | 0.62*** | 0.56*** | 0.48** | 0.39** | 0.47** | 0.74 |
| **Grip-Lift** |  |  |  |  |  |  |  |  |
| *OI* [ndom] | Stability | 0.70 | 0.65*** | 0.62*** | 0.30 | 0.34* | 0.63*** | 0.95 |
| *PI* [dom] | Stability | 0.81 | 0.63*** | 0.62*** | 0.35* | 0.34* | 0.67*** | 0.97 |
| *PI* [ndom] | Stability | 0.79 | 0.67*** | 0.67*** | 0.33* | 0.33* | 0.63*** | 0.97 |
| **Spiral Drawing** |  |  |  |  |  |  |  |  |
| *SPARC* | Smoothness | 0.75 | -0.71*** | -0.75*** | -0.43** | -0.48** | -0.63*** | n.a. |
| *Power_1-4 Hz_* | Smoothness | 0.82 | 0.73*** | 0.70*** | 0.38* | 0.46** | 0.75*** | n.a. |
| *acc_prc90-100_* | Speed | 0.72 | 0.60*** | 0.63*** | 0.23 | 0.33* | 0.66*** | n.a. |
| *speed_prc90-100_* | Speed | 0.78 | 0.71*** | 0.71*** | 0.32* | 0.38* | 0.78*** | n.a. |
| **Target Reaching** |  |  |  |  |  |  |  |  |
| *path_2D_* | Efficiency | 0.92 | 0.71*** | 0.75*** | 0.51** | 0.60** | 0.74*** | n.a. |
| *path_3D_* | Efficiency | 0.83 | 0.63*** | 0.71*** | 0.45* | 0.52** | 0.66*** | n.a. |
| *path_lr_* | Efficiency | 0.91 | 0.71*** | 0.71*** | 0.50** | 0.59** | 0.68*** | n.a. |
| *path_v_* | Efficiency | 0.78 | 0.57** | 0.63*** | 0.41* | 0.51** | 0.61*** | n.a. |
| *path_ap_* | Efficiency | 0.83 | 0.65*** | 0.66*** | 0.39* | 0.50** | 0.71*** | n.a. |
| *deviation_total_* | Efficiency | 0.93 | 0.63*** | 0.66*** | 0.46* | 0.51** | 0.65*** | n.a. |
| *dysmetria* | Endpoint | 0.85 | 0.70*** | 0.66*** | 0.45* | 0.59** | 0.71*** | n.a. |
| *hit rate* | Endpoint | 0.97 | -0.81*** | -0.79*** | -0.56** | -0.59** | -0.80*** | n.a. |
| *SPARC_2D_* | Smoothness | 0.89 | -0.64*** | -0.61*** | -0.32 | -0.41* | -0.69*** | n.a. |
| *SPARC_3D_* | Smoothness | 0.80 | -0.73*** | -0.73*** | -0.40* | -0.49** | -0.81*** | n.a. |
| *acc_2D,max_* | Speed | 0.81 | -0.74*** | -0.64*** | -0.50** | -0.39* | -0.66*** | n.a. |
| *acc_2D,mean_* | Speed | 0.93 | -0.79*** | -0.74*** | -0.54** | -0.49** | -0.73*** | n.a. |
| *latency_2D,acc_* | Speed | 0.85 | 0.79*** | 0.79*** | 0.57** | 0.50** | 0.70*** | n.a. |
| *acc_3D,max_* | Speed | 0.80 | -0.64*** | -0.56** | -0.45* | -0.29 | -0.53*** | n.a. |
| *acc_3D,mean_* | Speed | 0.92 | -0.77*** | -0.69*** | -0.53** | -0.46* | -0.72*** | n.a. |
| *latency_3D,acc_* | Speed | 0.85 | 0.70*** | 0.72*** | 0.43* | 0.34 | 0.64*** | n.a. |
| *dec_2D,mean_* | Speed | 0.94 | 0.70*** | 0.61*** | 0.53** | 0.51** | 0.64*** | n.a. |
| *latency_2D,dec_* | Speed | 0.87 | 0.80*** | 0.74*** | 0.47* | 0.40* | 0.73*** | n.a. |
| *dec_3D,mean_* | Speed | 0.93 | 0.68*** | 0.60** | 0.52** | 0.51** | 0.60*** | n.a. |
| *latency_3D,dec_* | Speed | 0.85 | 0.82*** | 0.77*** | 0.51** | 0.39* | 0.75*** | n.a. |
| *frequency* | Speed | 0.96 | -0.77*** | -0.75*** | -0.54** | -0.57** | -0.75*** | n.a. |
| *ITI* | Speed | 0.95 | 0.79*** | 0.76*** | 0.57** | 0.59** | 0.74*** | n.a. |
| *speed_2D,max_* | Speed | 0.88 | -0.71*** | -0.59** | -0.48** | -0.43* | -0.66*** | n.a. |
| *speed_2D,mean_* | Speed | 0.95 | -0.76*** | -0.74*** | -0.53** | -0.54** | -0.73*** | n.a. |
| *latency_2D,speed_* | Speed | 0.86 | 0.81*** | 0.80*** | 0.53** | 0.44* | 0.73*** | n.a. |
| *speed_3D,max_* | Speed | 0.87 | -0.71*** | -0.58** | -0.45* | -0.38* | -0.65*** | n.a. |
| *speed_3D,mean_* | Speed | 0.95 | -0.70*** | -0.66*** | -0.47* | -0.44* | -0.66*** | n.a. |
| *latency_3D,speed_* | Speed | 0.84 | 0.81*** | 0.80*** | 0.55** | 0.45* | 0.73*** | n.a. |
| *TD* | Speed | 0.90 | 0.65*** | 0.67*** | 0.48** | 0.53** | 0.61*** | n.a. |
| *latency_2D,acc,MAD_* | Variability | 0.85 | 0.82*** | 0.78*** | 0.49** | 0.44* | 0.78*** | n.a. |
| *latency_3D,acc,MAD_* | Variability | 0.85 | 0.75*** | 0.79*** | 0.48** | 0.41* | 0.72*** | n.a. |
| *latency_3D,dec,MAD_* | Variability | 0.92 | 0.78*** | 0.71*** | 0.43* | 0.43* | 0.75*** | n.a. |
| *latency_3D,dec,MAD_* | Variability | 0.92 | 0.77*** | 0.74*** | 0.46* | 0.42* | 0.77*** | n.a. |
| *ITI_MAD_* | Variability | 0.95 | 0.76*** | 0.77*** | 0.56** | 0.64*** | 0.73*** | n.a. |
| *path_2D,MAD_* | Variability | 0.90 | 0.70*** | 0.73*** | 0.51** | 0.55** | 0.79*** | n.a. |
| *path_3D,MAD_* | Variability | 0.87 | 0.66*** | 0.70*** | 0.46* | 0.53** | 0.74*** | n.a. |
| *path_V,MAD_* | Variability | 0.85 | 0.71*** | 0.75*** | 0.44* | 0.53** | 0.78*** | n.a. |
| *path_AP,MAD_* | Variability | 0.86 | 0.62*** | 0.61** | 0.34 | 0.42* | 0.71*** | n.a. |
| *latency_2D,speed,MAD_* | Variability | 0.87 | 0.74*** | 0.74*** | 0.45* | 0.43* | 0.71*** | n.a. |
| *latency_3D,speed,MAD_* | Variability | 0.87 | 0.74*** | 0.75*** | 0.44* | 0.40* | 0.71*** | n.a. |
| *TD_MAD_* | Variability | 0.93 | 0.65*** | 0.67*** | 0.37 | 0.46* | 0.68*** | n.a. |

3D/2D = 3D trajectory and virtual projection on 2D plane; dom/ndom = dominant/non-dominant hand;
Acc = Acceleration; Dec = Deceleration; ICC = intraclass correlation coefficient (trial 1 vs. trial 3);
IOI = inter-onset interval; IPI = inter-peak interval; ITI = inter-tap interval; MAD = median absolute deviation;
MED = median; OI = orientation index; PI = position index; STD = standard deviation; SPARC = Spectral Arc Length; TD = tap duration; *T_rise_* = duration of tap until peak force; *T_fall_* = duration of tap after peak force;
*p<0.05; **p<0.01; ***p<0.001.

**Supplement 5: Analysis of selected measures in healthy controls.**

| **Parameter** | **Feature** | **Sex**  **(Wilcoxon, *p*)** | **Age**  **(Spearman *rho)*** | **Height**  **(Spearman *rho*)** | **9HPT**  **(Spearman *rho*)** |
| --- | --- | --- | --- | --- | --- |
| **Finger Tapping** |  |  |  |  |  |
| Mean *TD* [dom] | Speed | 0.36 | 0.38** | 0.07 | 0.53*** |
| Mean *TD* [ndom] | Speed | 0.98 | 0.24 | -0.10 | 0.40** |
| Mean *T_fall_* [dom] | Speed | 0.18 | 0.40** | 0.12 | 0.58*** |
| Mean *T_fall_* [ndom] | Speed | 0.84 | 0.26 | -0.04 | 0.39** |
| *frequency* [dom] | Speed | 0.49 | -0.56*** | 0.12 | -0.59*** |
| *frequency* [ndom] | Speed | 0.34 | -0.41** | 0.28 | -0.55*** |
| Mean *IOI* [dom] | Speed | 0.52 | 0.55*** | -0.12 | 0.59*** |
| Mean *IOI* [ndom] | Speed | 0.33 | 0.41** | -0.29* | 0.54*** |
| Mean *IPI* [dom] | Speed | 0.36 | 0.48** | -0.16 | 0.51*** |
| Mean *IPI* [ndom] | Speed | 0.30 | 0.41** | -0.29* | 0.51*** |
| Mean *ITI* [dom] | Speed | 0.10 | 0.30* | -0.23 | 0.29 |
| Mean *ITI* [ndom] | Speed | 0.20 | 0.36* | -0.25 | 0.37** |
| STD *T_rise_* [ndom] | Variability | 0.93 | 0.04 | 0.01 | 0.19 |
| **Diadochokinesia** |  |  |  |  |  |
| *frequency* [ndom] | Speed | 0.56 | -0.37* | 0.24 | -0.52*** |
| Mean *IOI* [ndom] | Speed | 0.58 | 0.37** | -0.24 | 0.53*** |
| Mean *IPI* [ndom] | Speed | 0.61 | 0.35* | -0.23 | 0.52*** |
| Mean *ITI* [ndom] | Speed | 0.64 | 0.22 | -0.15 | 0.37** |
| STD *T_fall_* [ndom] | Variability | 0.26 | 0.26 | -0.20 | 0.41** |
| STD *IOI* [ndom] | Variability | 0.88 | 0.37* | -0.10 | 0.53*** |
| STD *IPI* [ndom] | Variability | 1.00 | 0.40** | -0.13 | 0.58*** |
| STD *ITI* [ndom] | Variability | 1.00 | 0.42** | -0.13 | 0.58*** |
| **Grip-Lift** |  |  |  |  |  |
| *orientation ind.* [ndom] | Stability | 0.32 | -0.29* | 0.01 | -0.03 |
| *position ind.* [dom] | Stability | 0.86 | -0.08 | -0.03 | 0.09 |
| *position ind.* [ndom] | Stability | 0.81 | -0.25 | -0.05 | 0.03 |
| **Spiral Drawing** |  |  |  |  |  |
| *SPARC* | Smoothness | 0.28 | -0.07 | 0.18 | -0.03 |
| *Power_1-4 Hz_* | Smoothness | 0.64 | 0.32* | -0.02 | 0.29* |
| *acc_prc90-100_* | Speed | 0.86 | -0.02 | 0.06 | -0.03 |
| *speed_prc90-100_* | Speed | 0.46 | 0.09 | 0.10 | 0.10 |
| **Target Reaching** |  |  |  |  |  |
| *path_2D_* | Efficiency | 0.09 | 0.31* | -0.18 | 0.25 |
| *path_3D_* | Efficiency | 0.06 | 0.31* | -0.12 | 0.11 |
| *path_lr_* | Efficiency | 0.03 | 0.31* | -0.17 | 0.17 |
| *path_v_* | Efficiency | 0.10 | 0.29 | -0.12 | 0.10 |
| *path_ap_* | Efficiency | 0.93 | 0.30* | 0.01 | 0.24 |
| *deviation_total_* | Efficiency | 0.29 | 0.47** | -0.20 | 0.48** |
| *dysmetria* | Endpoint | 0.69 | 0.20 | 0.03 | 0.26 |
| *hit rate* | Endpoint | 0.62 | -0.44** | 0.12 | -0.65*** |
| *SPARC_2D_* | Smoothness | 0.59 | -0.39** | -0.17 | -0.42** |
| *SPARC_3D_* | Smoothness | 0.98 | -0.24 | -0.06 | -0.24 |
| *acc_2D.max_* | Speed | 0.62 | -0.03 | -0.05 | -0.22 |
| *acc_2D.mean_* | Speed | 0.98 | -0.28 | 0.03 | -0.47** |
| *latency_2D.acc_* | Speed | 0.76 | 0.19 | -0.03 | 0.40** |
| *acc_3D.max_* | Speed | 0.69 | -0.01 | -0.02 | -0.22 |
| *acc_3D.mean_* | Speed | 0.96 | -0.26 | 0.03 | -0.45** |
| *latency_3D.acc_* | Speed | 0.88 | 0.27 | -0.05 | 0.39* |
| *dec_2D.mean_* | Speed | 0.67 | 0.44** | -0.12 | 0.55*** |
| *latency_2D.dec_* | Speed | 0.87 | 0.24 | -0.04 | 0.45** |
| *dec_3D.mean_* | Speed | 0.62 | 0.42** | -0.11 | 0.50*** |
| *latency_3D.dec_* | Speed | 0.51 | 0.23 | 0.05 | 0.44** |
| *frequency* | Speed | 0.47 | -0.44** | 0.16 | -0.55*** |
| *ITI* | Speed | 0.47 | 0.44** | -0.16 | 0.54*** |
| *speed_2D.max_* | Speed | 0.51 | -0.26 | -0.04 | -0.40** |
| *speed_2D.mean_* | Speed | 0.51 | -0.44** | 0.16 | -0.55*** |
| *latency_2D.speed_* | Speed | 0.81 | 0.16 | -0.03 | 0.39** |
| *speed_3D.max_* | Speed | 0.50 | -0.26 | -0.04 | -0.40** |
| *speed_3D.mean_* | Speed | 0.73 | -0.39** | 0.12 | -0.56*** |
| *latency_3D.speed_* | Speed | 0.72 | 0.12 | -0.02 | 0.40** |
| *TD* | Speed | 0.71 | 0.44** | -0.10 | 0.60*** |
| *latency_2D.acc.MAD_* | Variability | 0.84 | 0.03 | 0.01 | 0.33* |
| *latency_3D.acc.MAD_* | Variability | 0.46 | 0.17 | -0.09 | 0.29* |
| *latency_3D.dec.MAD_* | Variability | 0.43 | 0.27 | -0.10 | 0.40** |
| *latency_3D.dec.MAD_* | Variability | 0.92 | 0.12 | -0.02 | 0.38** |
| *ITI_MAD_* | Variability | 0.18 | 0.26 | -0.23 | 0.44** |
| *path_2D.MAD_* | Variability | 0.98 | 0.15 | 0.10 | 0.11 |
| *path_3D.MAD_* | Variability | 0.10 | 0.12 | -0.05 | 0.10 |
| *path_V.MAD_* | Variability | 0.04 | 0.15 | -0.16 | 0.14 |
| *path_AP.MAD_* | Variability | 0.66 | -0.15 | 0.05 | -0.08 |
| *latency_2D.speed.MAD_* | Variability | 0.70 | 0.19 | -0.05 | 0.35** |
| *latency_3D.speed.MAD_* | Variability | 0.94 | 0.35* | -0.03 | 0.37** |
| *TD_MAD_* | Variability | 0.73 | 0.37* | -0.11 | 0.51*** |

3D/2D = 3D trajectory and virtual projection on 2D plane; dom/ndom = dominant/non-dominant hand;
Acc = Acceleration; Dec = Deceleration; IOI = inter-onset interval; IPI = inter-peak interval; ITI = inter-tap interval; MAD = median absolute deviation; MED = median; STD = standard deviation; SPARC = Spectral Arc Length; TD = tap duration; *T_rise_* = duration of tap until peak force; *T_fall_* = duration of tap after peak force;

*p<0.05; **p<0.01; ***p<0.001.

**Supplement 6: Discrimination of measures between severity levels of upper limb ataxia**

| **Parameter** | **Feature** | **Mild** Median [IQR] | | **Moderate**  Median [IQR] | | **Severe**  Median [IQR] | | **Kruskal- Wallis, *p*** | | **Mild vs Mod**  (Wilcoxon, *p*) | | **Mod vs Sev**  (Wilcoxon*, p*) | |  |
| --- | --- | --- | --- | --- | --- | --- | --- | --- | --- | --- | --- | --- | --- | --- |
| **Finger Tapping** |  |  | |  | |  | |  | |  | |  | |  |
| Mean *TD* [dom] | Speed | 0.09 [0.04] | | 0.12 [0.06] | | 0.14 [0.04] | | < 0.001 | | 0.008 | | 0.155 | |  |
| Mean *TD* [ndom] | Speed | 0.11 [0.03] | | 0.15 [0.05] | | 0.18 [0.05] | | < 0.001 | | 0.003 | | 0.066 | |  |
| Mean *T_fall_* [dom] | Speed | 0.05 [0.02] | | 0.07 [0.03] | | 0.08 [0.02] | | < 0.001 | | 0.003 | | 0.379 | |  |
| Mean *T_fall_* [ndom] | Speed | 0.06 [0.01] | | 0.08 [0.04] | | 0.10 [0.02] | | < 0.001 | | 0.002 | | 0.054 | |  |
| *frequency* [dom] | Speed | 4.35 [1.23] | | 3.46 [0.76] | | 2.93 [0.55] | | < 0.001 | | 0.001 | | 0.112 | |  |
| *frequency* [ndom] | Speed | 4.17 [1.08] | | 2.97 [0.85] | | 2.62 [0.62] | | < 0.001 | | < 0.001 | | 0.054 | |  |
| Mean *IOI* [dom] | Speed | 0.23 [0.07] | | 0.29 [0.07] | | 0.34 [0.06] | | < 0.001 | | 0.001 | | 0.103 | |  |
| Mean *IOI* [ndom] | Speed | 0.24 [0.06] | | 0.34 [0.10] | | 0.38 [0.10] | | < 0.001 | | < 0.001 | | 0.066 | |  |
| Mean *IPI* [dom] | Speed | 0.22 [0.06] | | 0.27 [0.06] | | 0.31 [0.06] | | < 0.001 | | 0.001 | | 0.530 | |  |
| Mean *IPI* [ndom] | Speed | 0.23 [0.04] | | 0.31 [0.08] | | 0.34 [0.12] | | < 0.001 | | < 0.001 | | 0.143 | |  |
| Mean *ITI* [dom] | Speed | 0.13 [0.04] | | 0.18 [0.06] | | 0.20 [0.06] | | 0.002 | | 0.005 | | 0.451 | |  |
| Mean *ITI* [ndom] | Speed | 0.14 [0.04] | | 0.19 [0.06] | | 0.24 [0.06] | | < 0.001 | | < 0.001 | | 0.132 | |  |
| STD *T_rise_* [ndom] | Variability | 0.01 [0.00] | | 0.02 [0.01] | | 0.03 [0.02] | | < 0.001 | | 0.034 | | 0.009 | |  |
| **Diadochokinesia** |  |  | |  | |  | |  | |  | |  | |  |
| *frequency* [ndom] | Speed | 2.29 [0.67] | | 1.80 [0.56] | | 1.64 [0.42] | | < 0.001 | | 0.002 | | 0.209 | |  |
| Mean *IOI* [ndom] | Speed | 0.44 [0.12] | | 0.56 [0.17] | | 0.61 [0.17] | | < 0.001 | | 0.002 | | 0.194 | |  |
| Mean *IPI* [ndom] | Speed | 0.42 [0.11] | | 0.54 [0.14] | | 0.59 [0.14] | | < 0.001 | | 0.002 | | 0.121 | |  |
| Mean *ITI* [ndom] | Speed | 0.28 [0.06] | | 0.36 [0.07] | | 0.41 [0.12] | | < 0.001 | | 0.007 | | 0.049 | |  |
| STD *T_fall_* [ndom] | Variability | 0.04 [0.02] | | 0.06 [0.03] | | 0.07 [0.03] | | < 0.001 | | 0.021 | | 0.024 | |  |
| STD *IOI* [ndom] | Variability | 0.07 [0.04] | | 0.08 [0.06] | | 0.20 [0.13] | | < 0.001 | | 0.131 | | 0.002 | |  |
| STD *IPI* [ndom] | Variability | 0.07 [0.04] | | 0.09 [0.05] | | 0.20 [0.12] | | < 0.001 | | 0.034 | | 0.001 | |  |
| STD *ITI* [ndom] | Variability | 0.06 [0.04] | | 0.07 [0.04] | | 0.16 [0.09] | | < 0.001 | | 0.174 | | 0.001 | |  |
| **Grip-Lift** |  |  | |  | |  | |  | |  | |  | |  |
| *OI.* [ndom] | Stability | 2.88 [1.85] | | 6.06 [7.00] | | 8.13 [13.20] | | < 0.001 | | < 0.001 | | 0.492 | |  |
| *PI* [dom] | Stability | 0.57 [0.20] | | 1.14 [0.80] | | 1.46 [1.03] | | < 0.001 | | < 0.001 | | 0.258 | |  |
| *PI* [ndom] | Stability | 0.57 [0.24] | | 1.28 [0.99] | | 1.74 [1.44] | | < 0.001 | | < 0.001 | | 0.223 | |  |
| **Spiral Drawing** |  |  | |  | |  | |  | |  | |  | |  |
| *SPARC* | Smoothness | -2.34 [0.42] | | -4.79 [3.39] | | -5.94 [3.67] | | < 0.001 | | < 0.001 | | 0.060 | |  |
| *Power_1-4 Hz_* | Smoothness | 3.92 [1.86] | | 6.36 [3.79] | | 6.05 [5.78] | | < 0.001 | | < 0.001 | | 0.207 | |  |
| *acc_prc90-100_* | Speed | 6476 [3120] | | 11550 [9361] | | 15120 [8662] | | 0.001 | | 0.007 | | 0.097 | |  |
| *speed_prc90-100_* | Speed | 953 [203] | | 1120 [369] | | 1329 [456] | | < 0.001 | | 0.002 | | 0.044 | |  |
| **Target Reaching** |  |  | |  | |  | |  | |  | |  | |  |
| *path_2D_* | Efficiency | 31.4 [0.5] | | 32.0 [1.0] | | 36.4 [4.1] | | 0.001 | | 0.023 | | 0.009 | |  |
| *path_3D_* | Efficiency | 34.2 [2.4] | | 36.2 [1.9] | | 42.4 [9.9] | | 0.002 | | 0.023 | | 0.054 | |  |
| *path_lr_* | Efficiency | 14.5 [0.5] | | 15.0 [0.5] | | 17.4 [3.3] | | < 0.001 | | 0.055 | | 0.001 | |  |
| *path_v_* | Efficiency | 9.72 [4.34] | | 12.7 [2.6] | | 16.5 [9.1] | | 0.008 | | 0.046 | | 0.152 | |  |
| *path_ap_* | Efficiency | 26.4 [0.4] | | 26.5 [0.7] | | 28.4 [2.4] | | 0.016 | | 0.119 | | 0.094 | |  |
| **Parameter** | **Feature** | | **Mild** Median [IQR] | | **Moderate**  Median [IQR] | | **Severe**  Median [IQR] | | **Kruskal- Wallis, *p*** | | **Mild vs Mod**  (Wilcoxon, *p*) | | **Mod vs Sev**  (Wilcoxon*, p*) | |
| *deviation_total_* | Efficiency | | 503 [274] | | 807 [257] | | 1134 [400] | | 0.002 | | 0.046 | | 0.009 | |
| *Dysmetria* | Endpoint | | 0.24 [0.15] | | 0.37 [0.37] | | 1.26 [0.91] | | 0.011 | | 0.065 | | 0.072 | |
| *hit rate* | Endpoint | | 0.88 [0.22] | | 0.73 [0.14] | | 0.43 [0.12] | | < 0.001 | | 0.015 | | < 0.001 | |
| *SPARC_2D_* | Smoothness | | -1.44 [0.03] | | -1.45 [0.02] | | -1.61 [0.13] | | 0.001 | | 0.638 | | 0.001 | |
| *SPARC_3D_* | Smoothness | | -1.47 [0.03] | | -1.54 [0.09] | | -1.65 [0.14] | | 0.001 | | 0.046 | | 0.029 | |
| *acc_2D,max_* | Speed | | 658 [248] | | 473 [168] | | 313 [79] | | 0.003 | | 0.119 | | 0.029 | |
| *acc_2D,mean_* | Speed | | 259 [118] | | 187 [64] | | 113 [24] | | < 0.001 | | 0.023 | | 0.002 | |
| *latency_2D,acc_* | Speed | | 166 [65] | | 200 [53] | | 310 [66] | | < 0.001 | | 0.005 | | 0.001 | |
| *acc_3D,max_* | Speed | | 603 [278] | | 501 [205] | | 386 [75] | | 0.018 | | 0.158 | | 0.121 | |
| *acc_3D,mean_* | Speed | | 294 [111] | | 206 [69] | | 119 [17] | | 0.001 | | 0.046 | | 0.021 | |
| *latency_3D,acc_* | Speed | | 119 [39] | | 146 [37] | | 258 [47] | | < 0.001 | | 0.128 | | 0.001 | |
| *dec_2D,mean_* | Speed | | -169 [60] | | -131 [61] | | -96.0 [20.9] | | 0.003 | | 0.138 | | 0.029 | |
| *latency_2D,dec_* | Speed | | 443 [119] | | 525 [96] | | 710 [162] | | < 0.001 | | 0.055 | | 0.001 | |
| *dec_3D,mean_* | Speed | | -174 [72] | | -140 [59] | | -101 [17] | | 0.004 | | 0.180 | | 0.029 | |
| *latency_3D,dec_* | Speed | | 471 [120] | | 552 [149] | | 753 [148] | | < 0.001 | | 0.015 | | 0.006 | |
| *frequency* | Speed | | 1.03 [0.29] | | 0.85 [0.13] | | 0.58 [0.13] | | < 0.001 | | 0.039 | | 0.001 | |
| *ITI* | Speed | | 931 [294] | | 1151 [182] | | 1558 [376] | | < 0.001 | | 0.027 | | 0.001 | |
| *speed_2D,max_* | Speed | | 91.1 [17.2] | | 83.9 [16.7] | | 68.8 [10.4] | | 0.003 | | 0.262 | | 0.021 | |
| *speed_2D,mean_* | Speed | | 33.7 [10.1] | | 27.6 [5.4] | | 20.7 [2.9] | | < 0.001 | | 0.033 | | 0.004 | |
| *latency_2D,speed_* | Speed | | 276 [94] | | 363 [59] | | 516 [162] | | < 0.001 | | 0.005 | | 0.001 | |
| *speed_3D,max_* | Speed | | 94.2 [19.6] | | 84.8 [18.3] | | 72.0 [10.9] | | 0.005 | | 0.232 | | 0.040 | |
| *speed_3D,mean_* | Speed | | 36.6 [9.4] | | 31.6 [8.8] | | 24.4 [1.3] | | 0.002 | | 0.103 | | 0.009 | |
| *latency_3D,speed_* | Speed | | 277 [96] | | 364 [46] | | 494 [74] | | < 0.001 | | 0.003 | | 0.001 | |
| *TD* | Speed | | 144 [59] | | 187 [34] | | 421 [180] | | < 0.001 | | 0.119 | | < 0.001 | |
| *latency_2D,acc,MAD_* | Variability | | 20.8 [10.1] | | 33.7 [21.9] | | 105 [24] | | < 0.001 | | 0.012 | | < 0.001 | |
| *latency_3D,acc,MAD_* | Variability | | 20.1 [17.2] | | 36.9 [28.5] | | 92.0 [30.7] | | < 0.001 | | 0.033 | | < 0.001 | |
| *latency_3D,dec,MAD_* | Variability | | 34.7 [12.9] | | 45.5 [14.2] | | 123 [10] | | < 0.001 | | 0.055 | | < 0.001 | |
| *latency_3D,dec,MAD_* | Variability | | 41.7 [8.5] | | 59.4 [16.7] | | 143 [35] | | < 0.001 | | 0.033 | | < 0.001 | |
| *ITI_MAD_* | Variability | | 53.5 [34.9] | | 89.2 [30.2] | | 210 [110] | | < 0.001 | | 0.019 | | < 0.001 | |
| *path_2D,MAD_* | Variability | | 0.46 [0.18] | | 0.62 [0.25] | | 1.53 [0.76] | | < 0.001 | | 0.027 | | 0.006 | |
| *path_3D,MAD_* | Variability | | 0.62 [0.27] | | 1.02 [0.65] | | 2.13 [1.68] | | 0.001 | | 0.055 | | 0.014 | |
| *path_V,MAD_* | Variability | | 0.94 [0.52] | | 1.13 [0.67] | | 2.31 [0.47] | | 0.001 | | 0.027 | | 0.009 | |
| *path_AP,MAD_* | Variability | | 0.33 [0.19] | | 0.42 [0.30] | | 1.03 [0.51] | | 0.003 | | 0.065 | | 0.054 | |
| *latency_2D,speed,MAD_* | Variability | | 18.8 [13.7] | | 30.2 [11.1] | | 86.8 [25.3] | | < 0.001 | | 0.012 | | < 0.001 | |
| *latency_3D,speed,MAD_* | Variability | | 21.5 [13.9] | | 32.3 [16.3] | | 95.1 [17.6] | | < 0.001 | | 0.012 | | < 0.001 | |
| *TD_MAD_* | Variability | | 20.1 [15.5] | | 28.5 [14.9] | | 86.1 [50.2] | | < 0.001 | | 0.328 | | < 0.001 | |

**Mild**: SARAul = 0-2 points; **Moderate**: SARAul = >2-4 points; **Severe**: SARAul = >4-6 points

3D/2D = 3D trajectory and virtual projection on 2D plane; dom/ndom = dominant/non-dominant hand;
Acc = Acceleration; Dec = Deceleration; IOI = inter-onset interval; IPI = inter-peak interval; ITI = inter-tap interval; MAD = median absolute deviation; OI = orientation index; PI = position index; STD = standard deviation; SPARC = Spectral Arc Length; TD = tap duration; *T_rise_* = duration of tap until peak force; *T_fall_* = duration of tap after peak force; *p<0.05; **p<0.01; ***p<0.001.

**Supplement 7: Measures with strong correlations to upper limb impairment in mild ataxia (SARA≤ 10).**

| **Parameter** | **Feature** | **AUC_AvC_** | **rho_SARA_** | **rho_SARAul_** | **rho_ADL_** | **rho_ADLul_** | **rho_9HPT_** |
| --- | --- | --- | --- | --- | --- | --- | --- |
| **Finger Tapping** |  |  |  |  |  |  |  |
| *frequency* [dom] | Speed | 0.83 | -0.37 | -0.26 | -0.7** | -0.53* | -0.34 |
| Mean *IOI* [dom] | Speed | 0.83 | 0.34 | 0.26 | 0.68** | 0.5* | 0.34 |
| Mean *IPI* [dom] | Speed | 0.84 | 0.34 | 0.26 | 0.64** | 0.39 | 0.35 |
| Mean *IPI* [ndom] | Speed | 0.79 | 0.48* | 0.49 | 0.61** | 0.34 | 0.44 |
| Mean *ITI* [ndom] | Speed | 0.74 | 0.44 | 0.41 | 0.61** | 0.53* | 0.37 |
| **Diadochokinesia** |  |  |  |  |  |  |  |
| *frequency* [ndom] | Speed | 0.87 | -0.30 | -0.28 | -0.62** | -0.41 | -0.26 |
| Mean *IOI* [ndom] | Speed | 0.87 | 0.28 | 0.27 | 0.61** | 0.40 | 0.27 |
| **Spiral Drawing** |  |  |  |  |  |  |  |
| *acc_MAD_* | Smoothness | **0.75** | **0.62**** | **0.37** | **0.47*** | **0.46*** | **0.69**** |
| *acc_MED_* | Speed | 0.76 | 0.60** | 0.31 | 0.42 | 0.37 | 0.64** |
| *acc_prc90-100_* | Speed | **0.75** | **0.61**** | **0.37** | **0.47*** | **0.45*** | **0.67**** |
| **Target Reaching** |  |  |  |  |  |  |  |
| *path_2D_* | Efficiency | 0.90 | 0.50 | 0.52 | 0.33 | 0.44 | 0.73** |
| *path_ap_* | Efficiency | 0.74 | 0.57* | 0.52 | 0.28 | 0.51 | 0.74** |
| *path_2D.MAD_* | Variability | **0.85** | **0.54*** | **0.37** | **0.54*** | **0.45** | **0.76**** |
| *path_3D.MAD_* | Variability | 0.83 | 0.55* | 0.39 | 0.44 | 0.51 | 0.70** |
| *path_LR.MAD_* | Variability | 0.88 | 0.54* | 0.53 | 0.29 | 0.33 | 0.82** |
| *path_V.MAD_* | Variability | 0.75 | 0.56* | 0.49 | 0.23 | 0.44 | 0.70** |
| *path_AP.MAD_* | Variability | 0.77 | 0.43 | 0.10 | 0.42 | 0.25 | 0.60* |
| *dtw_2D_* | Variability | 0.81 | 0.17 | 0.09 | -0.04 | -0.02 | 0.71** |
| *dtw_3D_* | Variability | 0.81 | 0.32 | 0.26 | -0.07 | -0.03 | 0.75** |

3D/2D = 3D trajectory and virtual projection on 2D plane; dom/ndom = dominant/non-dominant hand;
Acc = Acceleration; IOI = inter-onset interval; IPI = inter-peak interval; ITI = inter-tap interval;
MED = median; MAD = median absolute deviation; SPARC = Spectral Arc Length.
Bold correlations highlight measures that passed selection criteria. *p<0.05; **p<0.01; ***p<0.001.
